# Melanocortin 1 receptor attenuates early brain injury following subarachnoid hemorrhage by controlling mitochondrial metabolism *via* AMPK/SIRT1/PGC-1α pathway in rats

**DOI:** 10.7150/thno.49426

**Published:** 2021-01-01

**Authors:** Weilin Xu, Jun Yan, Umut Ocak, Cameron Lenahan, Anwen Shao, Jiping Tang, Jianmin Zhang, John H. Zhang

**Affiliations:** 1 Department of Neurosurgery, The Second Affiliated Hospital, Zhejiang University School of Medicine, Hangzhou 310009, Zhejiang, China.; 2 Department of Physiology & Pharmacology Loma Linda University, Loma Linda, CA 92350, USA.; 3 Department of Neurosurgery Loma Linda University, Loma Linda, CA 92350, USA.; 4 Department of Neurosurgery, Guangxi Medical University Cancer Hospital, 71 He Di Road, Nanning, 530021, Guangxi Zhuang Autonomous Region, China.; 5 Department of Emergency Medicine, Bursa Yuksek Ihtisas Training and Research Hospital, University of Health Sciences, Bursa 16310, Turkey.; 6 Department of Emergency Medicine, Bursa City Hospital, Bursa 16110, Turkey.; 7 Burrell College of Osteopathic Medicine, New Mexico State University, Las Cruces, NM, USA.; Weilin Xu and Jun Yan have equally contributed to this work as co-first authors.

**Keywords:** melanocortin 1 receptor, mitochondrial fission, oxidative stress, peroxisome proliferator-activated receptor gamma coactivator 1-alpha, subarachnoid hemorrhage

## Abstract

Mitochondria-mediated oxidative stress and apoptosis contribute greatly to early brain injury (EBI) following subarachnoid hemorrhage (SAH). This study hypothesized that activation of melanocortin 1 receptor (MC1R), using BMS-470539, attenuates EBI by controlling mitochondrial metabolism after SAH.

**Methods:** We utilized BMS-470539, MSG-606, selisistat, and PGC-1α to verify the neuroprotective effects of MC1R. We evaluated short- and long-term neurobehavior after SAH. Western blotting, immunofluorescence, and Golgi staining techniques were performed to assess changes in protein levels.

**Results:** The results of western blotting suggested that the expression of SIRT1 and PGC-1α were increased, reaching their peaks at 24 h following SAH. Moreover, BMS-470539 treatment notably attenuated neurological deficits, and also reduced long-term spatial learning and memory impairments caused by SAH. The underlying neuroprotective mechanisms of the BMS-470539/MC1R system were mediated through the suppression of oxidative stress, apoptosis, and mitochondrial fission by increasing the levels of SIRT1, PGC-1α, UCP2, SOD, GPx, Bcl-2, cyto-Drp1, and ATP, while decreasing the levels of cleaved caspase-3, Bax, mito-Drp1, ROS, GSH/GSSG, and NADPH/NADP+ ratios. The neuroprotective effects of the BMS-470539/MC1R system were significantly abolished by MSG-606, selisistat, and PGC-1α siRNA.

**Conclusions:** The activation of MC1R with BMS-470539 significantly attenuated EBI after SAH by suppressing the oxidative stress, apoptosis, and mitochondrial fission through the AMPK/SIRT1/PGC-1α signaling pathway.

## Introduction

Mitochondria are one of the most important organelles in cells, and are critical for energy production within the central nervous system (CNS) 1. In addition, mitochondria are involved in several processes, such as cell differentiation, apoptosis, and reactive oxygen species (ROS) generation 2, 3. Under normal circumstances, the production and elimination of ROS in cells maintain a dynamic balance. However, mitochondrial dysfunction increases the level of ROS 4, 5. Besides, given that mitochondrial DNA has no histone protection; it is extremely vulnerable to damage by ROS. Once mitochondria are damaged, cytochrome C is released, further activating apoptotic factors, including caspase-9, caspase-3, etc., thereby inducing apoptosis 6, 7. Recent studies have found that mitochondrial fusion and fission play important roles in maintaining mitochondrial stability 8, 9. On the other hand, excessive mitochondrial fission is harmful, as it increases the production of ROS. Therefore, suppression of mitochondrial fission is an important method in reducing the production of ROS and neuronal apoptosis induced by mitochondrial dysfunction.

Subarachnoid hemorrhage (SAH) induces a series of pathophysiological processes, including inflammation, oxidative stress, apoptosis, and cell electrophysiological disorders 10. In recent years, it has been suggested that early brain injury (EBI) is the main contributor to the poor prognosis associated with SAH. Shortly after the induction of SAH, EBI induces mitochondrial dysfunction, which contributes to the oxidative stress and apoptosis 11. Therefore, one of the keys in decreasing EBI is uncovering novel methods to reduce intracellular ROS and maintaining normal mitochondrial activity by reducing neuronal apoptosis. The PGC-1α (peroxisome proliferator-activated receptor gamma coactivator 1-alpha) is involved in cellular energy 12, and recently, it has been found to play a key role in regulating mitochondrial production and anti-oxidative stress 13, 14. Li *et al.* has indicated that PGC-1α enters the nucleus to promote the expression of antioxidant enzymes, such as superoxide dismutase (SOD), catalase, and glutathione peroxidase (GPx), to reduce intracellular ROS levels, thereby protecting normal mitochondrial function 15. In addition, Cordani *et al.* has found that PGC-1α can reduce the generation of ROS by regulating the level of UCP2, and by protecting mitochondria from ROS-induced damage 16. Moreover, Ding *et al.* has reported that PGC-1α is associated with decreased mitochondrial fission by suppressing the activity of Drp1 17. However, the role of PGC-1α in SAH has been rarely studied so far.

To be fully activated, PGC-1α requires both AMPK (Adenosine 5'-monophosphate (AMP)-activated protein kinase) phosphorylation and SIRT1 (silent information regulator 1) acetylation 18. AMPK is a key molecule in the regulation of bioenergy metabolism, but it also plays an important role in the regulation of apoptosis and oxidative stress 19, 20. Yan *et al.* has demonstrated that AMPK reduces ROS production through the activation of PGC-1α 21. In addition, AMPK can indirectly activate SIRT1 by regulating the level of NAD+ 22, which is an NAD+-dependent protein deacetylase that participates in neuroprotection by reducing oxidative stress. SIRT1 activation reportedly improves mitochondrial function and attenuates brain injury by increasing the level of PGC-1α 23. Therefore, pharmacological regulation of the AMPK/SIRT1/PGC-1α signaling pathway may be of importance in reducing mitochondria-mediated oxidative stress and apoptosis following SAH.

The melanocortin receptor-1 (MC1R), a member of the MCR family, is a type of G-protein coupled receptor 24. In our previous study, we have demonstrated that MC1R activation can increase AMPK activity 25. Torrens *et al.* has indicated that MC1R can reduce intracellular levels of ROS by regulating PGC-1α activity, thereby reducing oxidative stress-induced apoptosis 25. However, the exact mechanisms by which MC1R activates PGC-1α remain unknown. Collectively, we hypothesize that the activation of MC1R can regulate the AMPK/SIRT1/PGC-1α signaling pathway, which successively reduces the levels of intracellular oxidative stress, protects normal mitochondrial metabolism, and diminishes neuronal apoptosis, thereby alleviating EBI after SAH.

## Materials and methods

### Animals

All experiments in this study were conducted according to the protocols proposed by the Institutional Animal Care and Use Committee (IACUC) at Loma Linda University. All operations were performed according to the NIH guidelines. In detail, we used 280-330 g male Sprague-Dawley rats (Indianapolis, IN) for this study. The rats were kept in constant temperature (22 ± 1 °C) and humidity (60 ± 5%) on a 12 h day/night cycle, with food and water provided *ad libitum*.

### Experimental design

The study consists of nine different experiments as shown in Figure S1. The detailed information regarding the number and distribution of animals used in this study is presented in Table S1.

**Experiment 1**. The levels of SIRT1 and PGC-1α proteins were determined in sham and SAH groups at different time-points (3, 6, 12, 24, and 72 h). We sampled the ipsilateral/left cerebral cortex for western blotting. In addition, double immunofluorescence staining was applied to explore the expression of MC1R in three major types of neural cells (neurons, microglia and astrocytes) in sham and SAH (24 h) groups.

**Experiment 2.** Three different concentrations of BMS-470539 (sc-362716A, Santa Cruz) were prepared in order to identify the best dose with greatest neuroprotective effects. The preparation of BMS-470539 was based on a previous study 26. BMS-470539 was administrated intranasally (i.n.) 1 h after SAH. Neurobehavioral tests, including modified Garcia score and beam balance tests were conducted at 24 h after SAH.

**Experiment 3.** Based on the better neurological outcomes observed in the previous step, the medium dosage (160 μg/kg, 10 μl) was selected for the following studies. Rats were randomly divided into three groups to evaluate long-term neurological function: sham, SAH + vehicle, and SAH + BMS-470539. In detail, we conducted rotarod tests on the 1^st^, 2^nd^, and 3^rd^ weeks following SAH. In addition, we performed Morris water maze test on days 21 to 25 after SAH. Besides, we randomly chose three rats in each group for Nissl staining. In order to evaluate the anti-apoptotic and anti-oxidative stress effects of BMS-470539 after SAH rats, we set another three groups of rats. Western blotting, evaluation with transmission electron microscopy (TEM), ROS and ATP assays, as well as immunofluorescence staining were conducted 24 h after SAH.

**Experiment 4.1** To study the role of MC1R-mediated anti-oxidative stress and anti-apoptotic effects, MSG-606 (5954, R&D systems), the specific antagonist of MC1R was applied in this experiment. MSG-606 was administered *via* intracerebroventricular (i.c.v.) administration 1 h before SAH. Neurological functions were evaluated 24 h after SAH. We also collected the ipsilateral/left cerebral cortex of the subjects for western blotting, evaluation of ROS and ATP, and immunofluorescence staining.

**Experiment 4.2** To assess the role of SIRT1 in MC1R-mediated anti-oxidative stress and anti-apoptosis pathways, the selective SIRT1 inhibitor, selisistat (Selleck, EX527), was administrated *via* i.c.v. administration 1 h before SAH. Neurological testing was conducted at 24 h after SAH. Western blotting, ROS and ATP assays, as well as immunofluorescence staining were conducted 24 h after SAH.

**Experiment 4.3** To evaluate the relationship between AMPK and SIRT1, the specific AMPK inhibitor, dorsomorphin (Sigma-Aldrich), was administrated *via* i.c.v. administration 30 min before SAH. Western blotting, ROS and ATP assays, as well as immunofluorescence staining were conducted 24 h after SAH.

**Experiment 4.4** To evaluate the role of PGC-1α in MC1R-mediated anti-oxidative stress and anti-apoptosis pathway, PGC-1α siRNA (Santa Cruz) was administered *via* i.c.v. administration 48 h before SAH. Neurological functions were evaluated 24 h after SAH. Western blotting, ROS and ATP assays, and immunofluorescence staining were performed 24 h after SAH.

**Experiment 5.** To evaluate the effects of PGC-1α on mitochondrial genesis and fission, a specific inhibitor of Drp1, Mdivi-1 (MedChem Express), was administered *via* intravenous (i.v.) administration 30 min before SAH. Neurological functions were evaluated 24 h after SAH. Western blotting, evaluation of ROS, ATP, glutathione/oxidized glutathione (GSH/GSSG) and NADPH/NADP^+^, and immunofluorescence staining were conducted 24 h after SAH.

**Experiment 6.** We used naive rats to evaluate knockout efficiency of PGC-1α siRNA by i.c.v. injection of PGC-1α siRNA. We sampled ipsilateral/left cerebral cortex for western blotting 24 h after SAH.

### SAH animal model

We performed a widely used and well-recognized endovascular perforation SAH model 27 for this study. Briefly, the rats were anesthetized with 5% isoflurane and anesthesia was maintained with 3% isoflurane on a ventilator. We then exposed the carotid artery and its bifurcation. Then, a 4-0 sharpened nylon suture was inserted along the internal carotid artery. Finally, a perforation was made in the bifurcation of the anterior and middle cerebral arteries (Figure S2A). Detailed steps of the procedure were shown in supplemental materials. The degree of SAH was assessed using a new grading system as previously described 28.

### Drug administration and intracerebroventricular injection

BMS-470539 was administered intranasally, and the dosage was chosen according to a previous report 26. MSG-606 (1 nmol/μl, 6 μl) and selisistat were administered *via* i.c.v. injection 1 h before SAH. Dorsomorphin (0.1 μmol, 10 μl) was administered *via* i.c.v. injection 30 min before SAH. Mdivi-1(1.2 mg/kg) was given by i.v. injection 30 min before SAH 29-32. The rat PGC-1α siRNA, RISP siRNA, and the same volume of scramble siRNA (negative control) (abm inc., USA) were injected into the right ventricle (3.5 mm depth below the skull) 48 h before the induction of SAH. Detailed steps of the i.c.v injection were explained in supplemental materials.

### Mortality, SAH grade and short-term neurological assessment

We decided to evaluate neurological function at 24 h, given the peaked expression of SIRT1 and PGC-1α at 24 h after SAH. Neurological functions (modified Garcia scoring system 28 and beam balance test 33) were assessed by an independent researcher. Detailed information of neurobehavior evaluation is presented in Tables S2 and S3.

### Long-term neurological evaluation

We performed rotarod tests to assess long-term neurobehavior in the 1^st^, 2^nd^, and 3^rd^ weeks, and Morris water maze test to evaluate the spatial learning and memory abilities on days 21 to 25 following SAH as previously reported 30. Detailed information pertaining to the Morris water maze test is depicted in Table S4.

### Western blot analysis

We performed western blotting according to a previous report 34. The primary antibodies used include: MC1R (1:500, ThermoFisher, PA5-75342), SIRT1 (1:5000, Abcam ab110304), PGC-1α (1:1000, Abcam ab54481), UCP2 (1:500, Abcam ab67241), GPx (1:500, Abcam ab59524), SOD (1:500, Abcam ab9722), cleaved caspase-3 (1:500, Abcam ab49822), Bcl-2 (1:500, Abcam ab59348), Bax (1:3000, Abcam ab32503), Drp1 (1:1000, Abcam ab184247), p-AMPK (1:1000, Cell Signaling, #2535), AMPK (1:2000, Cell Signaling, #5832), and β-actin (1: 1000, Abcam ab8227). Detailed procedures of western blotting are presented in supplemental materials.

### Immunofluorescence staining (TUNEL, DHE, and Fluoro-Jade C) staining

The primary antibodies used include: MC1R (1:50, ThermoFisher, PA5-75342), NeuN (1:100, Abcam ab104224), glial fibrillary acidic protein (GFAP) (1:100, Abcam ab53554), and Iba-1 (1:100, Abcam ab48004). A detailed description of the immunofluorescence staining is provided in the supplemental materials. We performed terminal deoxynucleotidyl transferase-dUTP nick end labeling (TUNEL) staining to detect apoptotic cell death according to the manufacturer's protocol (Roche Inc, Basel, Switzerland). Fluoro-Jade C (FJC) staining was conducted to identify the degenerating neurons using the FJC staining kit (Biosensis, USA) according to the manufacturer's instructions. Dihydroethidium (DHE) staining was conducted to assess the level of ROS using the DHE staining kit (DHE, Thermo Fisher Scientific, USA) according to the manufacturer's instructions. TUNEL-, DHE-, and FJC-positive neurons were counted in the ipsilateral/left cerebral cortex by an independent researcher.

### Nissl staining

We performed Nissl staining as previously described 35. Detailed procedures of Nissl staining can be found in the supplemental materials. The agent we used was 0.5% cresyl violet (Sigma-Aldrich, St. Louis, MO, USA). The results were assessed by an independent researcher.

### Golgi staining

We performed Golgi staining using the FD Rapid GolgiStain™ (Columbia, USA) Kit as previously described 36. The procedures were conducted according to the manufacturer's protocol, which can be found in the supplemental materials.

### ROS and ATP assay

We performed the ROS and ATP assays based on the instructions of the ROS assay kit (JianCheng, China) and ATP assay kit (Beyotime, SHANGHAI, CHINA), respectively. Detailed procedures were reported by our recent study 37.

### GSH/GSSG, NADPH/NADP^+^ evaluation

We performed GSH/GSSG and NADPH/NADP^+^ assays using the GSSG assay kit (Beyotime, Shanghai, China) and the NADPH/NADP^+^ quantification kit (Sigma, St. Louis, MO, Cambridge, UK), respectively.

### Transmission electron microscopy (TEM)

TEM evaluation was conducted according to a previous report 31. Briefly, after euthanasia, the left cortical regions of the rats were sampled and sliced into 100-nm sections. After being stained with 4% uranyl acetate (20 min) and 0.5% lead citrate (5 min), the sections were visualized using the TEM (Philiphs Tecnai 10). A detailed description of the TEM evaluation can be found in the supplemental materials.

### Statistical analysis

We organized and displayed the data by mean ± standard deviation (SD). First, we conducted power analysis based on α = 0.05, the number of groups by the SPSS 22.0 software (IBM, USA), and G*power 3.0 (Faul, Erdfelder, Lang, & Buchner), with a power of 0.85 or higher indicating reliable evidence. We also calculated the sample size based on a method reported by Charan *et al.* 38. Then, the normality of the data was assessed. If the data met the requirement of satisfied normality and homogeneity of variance, one-way analysis of variance (ANOVA), followed by Tukey's post-hoc test for multiple comparisons between different groups was used. For the data that failed the normality test, non-parametric statistics (Kruskal-Wallis H) were applied. Additionally, two-way repeated measures ANOVA were applied to analyze the data of long-term neurological functions. Statistical significance was defined as *P* < 0.05. GraphPad Prism (GraphPad Software, San Diego, CA, USA) was applied to analyze the data.

## Results

### Physiological data

Physiological parameters, including body temperature, blood pressure, heart rate, blood glucose, PO_2_, and PCO_2_, were recorded during the surgery. No significant differences were observed between groups (Table S5).

### Mortality Rates and SAH Grade

A total of 331 rats were used in the present study, including dead and excluded animals. Among these, 43 were in the sham group, whereas 240 rats were subjected to SAH. Thirty-three rats died, and nine rats were excluded, as their SAH grades were less than 7 (Table S1). Among the dead animals, 22 died immediately after surgery. Of them, 17 died due to severe SAH, 4 died due to pulmonary hemorrhage, and 1 died due to an anesthesia complication. In addition, 11 rats died during the following periods: 9 rats died within 24 h after surgery (6 rats died due to severe SAH, 1 rat died due to throat edema and pulmonary injury, 2 rats died of unknown reason), and 2 rats died on day 2 and day 5 following the surgery due to severe SAH. We then added new animals to replace the excluded rats to ensure that there were six rats per group. Besides, 83 rats were shared among different groups according to the “Three Rs principle (replacement, reduction, and refinement)” 39. Blood clots were observed primarily around the circle of Willis and the ventral brain stem after the induction of SAH. We elected to proceed with the left cortical region for this study (Figure S2A). There was no statistically significant difference in SAH grades among different SAH groups (Figure S2B).

### Temporal patterns of SIRT1 and PGC-1α were assessed after SAH

**Figure 1:**
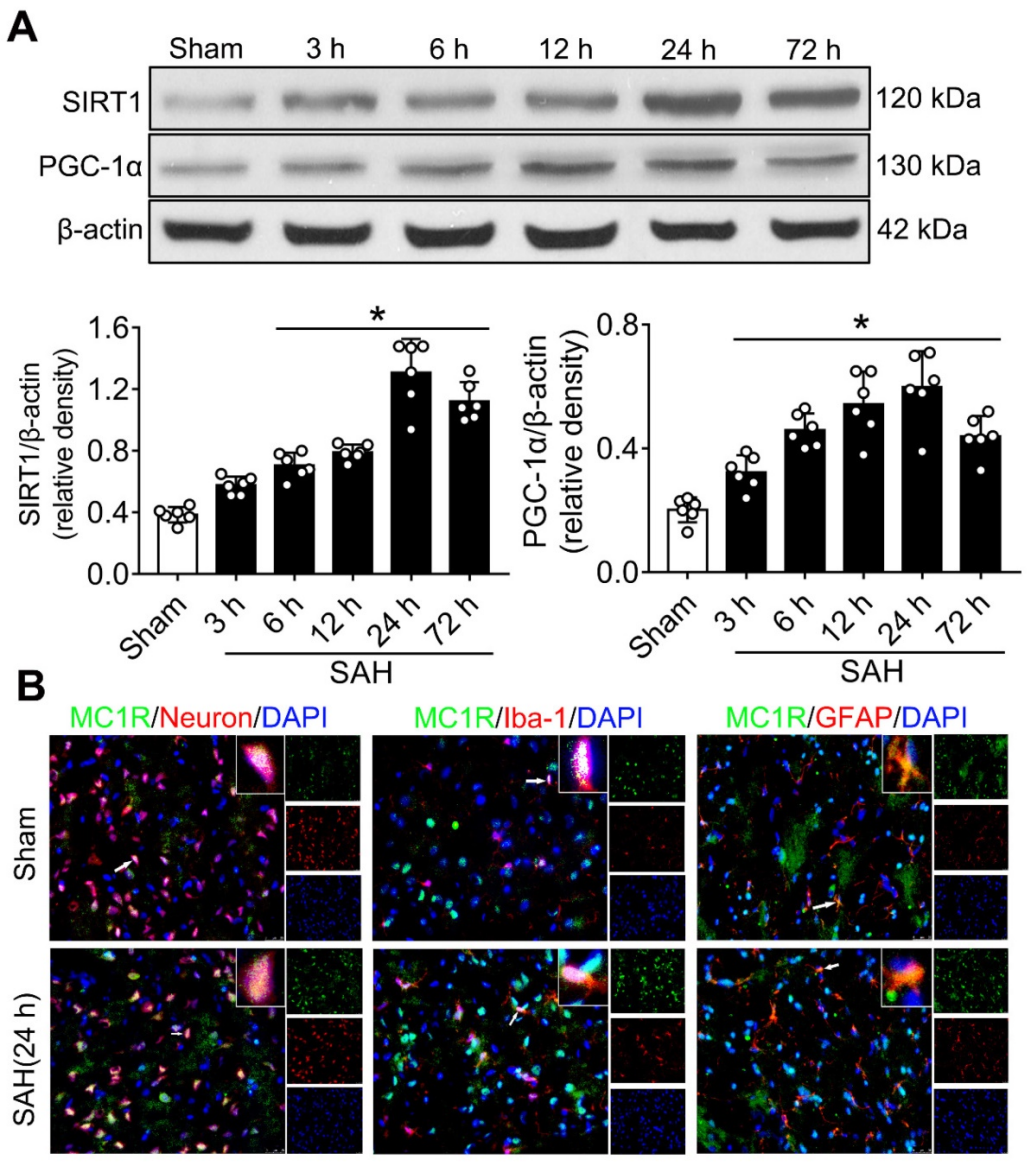
The expression of MC1R, SIRT1 and PGC-1α. (A) Representative western blot images and quantitative analyses of SIRT1 and PGC-1α of the time course study in samples obtained from the left hemisphere after SAH; n = 6 for each group. Data were shown as means ± SD, and were compared by 1-way ANOVA followed by the Tukey post-hoc test. *p < 0.05 *vs.* sham; (B) Representative microphotographs of immune-fluorescence staining, showing colocalization of MC1R (green) with NeuN, iba-1, and GFAP (all red) 24 h in the sham and SAH (24 h) groups. (n = 2 for each group). Scale bar = 50 μm.

Western blotting was conducted to evaluate the protein expression of SIRT1 and PGC-1α in sham and SAH animals at different time points (3 h, 6 h, 12 h, 24 h, and 72 h) after SAH. The levels of SIRT1 and PGC-1α began to increase at 3 h, and peaked at 24 h after SAH (Figure 1A). There was a significant difference between the sham and 24 h groups (P < 0.05; Figure 1A).

### Cellular location of MC1R

Double immunofluorescence staining showed that the expression of MC1R can be found in neurons (NeuN), microglia (Iba-1), and astrocytes (GFAP), and its expression was notably increased after SAH. Moreover, MC1R-positive cells were mainly located in neurons and microglia (Figure 1B).

### BMS-470539 administration improved both short- and long-term neurobehavior after SAH

**Figure 2:**
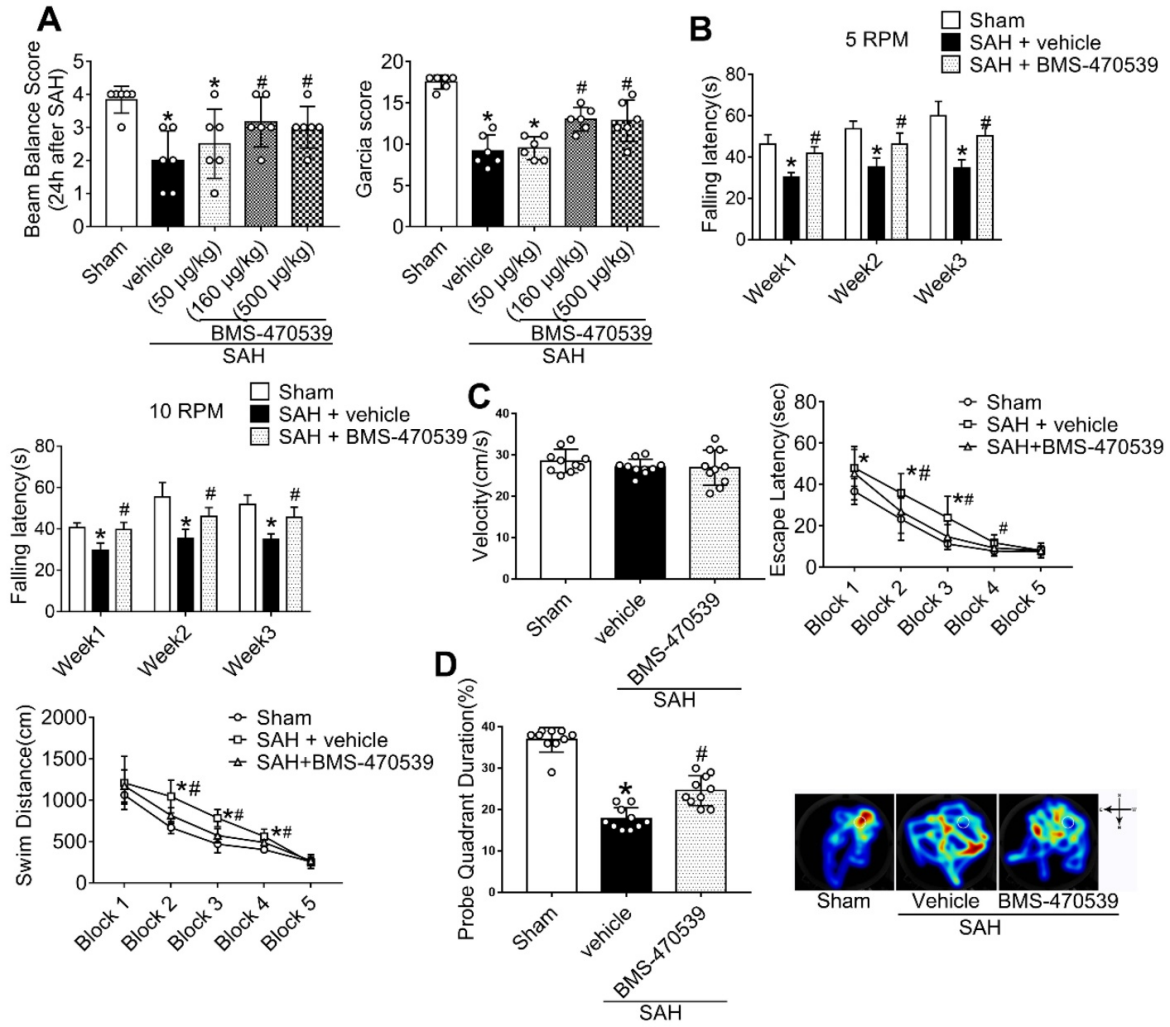
Effects of BMS-470539 on short-term and long-term behaviors of rats subjected to SAH. (A) Beam balance and modified Garcia scores for each group, n = 6 per group; (B) Rotarod test of 5 RPM and 10 RPM, n = 10 per group. (C) Velocity, escape latency, and swim distance of water maze test, n = 10 per group; (D) Probe quadrant duration and typical traces of water maze test, n = 10 per group. Data of beam balance scores were shown as the medians with interquartile range, and were compared using the Kruskal-Wallis test followed by the Dunn post-hoc test. Data of rotarod test, escape latency and swim distance were shown as means ± SD and compared by 2-way ANOVA test. Other data were shown as means ± SD, and were compared using 1-way ANOVA followed by the Tukey post-hoc test. *p < 0.05 *vs.* sham, &*p <* 0.05 *vs.* SAH + vehicle.

In short-term neurobehavior evaluation, scores of both modified Garcia and beam balance tests were significantly decreased in the SAH + vehicle group compared to the sham group. However, the administration of medium (160 μg/kg) and high (500 μg/kg) dosages of BMS-470539 significantly improved neurobehavior when compared with the SAH + vehicle group (P < 0.05; Figure 2A), while a low dosage (50 μg/kg) of BMS-470539 failed to improve the neurobehavior (Figure 2A).

In long-term neurobehavior evaluation (Table S4), rotarod tests were conducted in the 1^st^, 2^nd^, and 3^rd^ weeks following SAH. The results revealed that rats in the SAH group had shorter falling latency than that of the sham group (P < 0.05; Figure 2B) at both 5 and 10 RPMs. However, rats receiving BMS-470539 remained on the roller for a longer duration in the 1^st^, 2^nd^, and 3^rd^ weeks when compared with SAH + vehicle group (P < 0.05; Figure 2B).

In addition, the Morris water maze test was conducted on days 21 to 25 after SAH. There was no significant difference in velocity between different groups (Figure 2C). However, the rats that underwent SAH induction showed a significant decrease in escape latency in blocks 1, 2, and 3, and swam a longer distance to find the platform in blocks 2, 3, and 4 than the rats in sham group. Moreover, the rats receiving BMS-470539 had improved escape latency in blocks 2 and 3, and shorter swimming distance to find the platform in blocks 2, 3, and 4 compared to the SAH + vehicle group (P < 0.05; Figure 2C). For probe trials, the results showed that the rats receiving SAH induction stayed for a shorter duration (18%) in the target quadrant compared to the sham group (37%), while the administration of BMS-470539 significantly reversed the results (25%, Figure 2D).

### BMS-470539 exerted its neuroprotective effects *via* the suppression of neuronal apoptosis and oxidative stress after SAH

**Figure 3:**
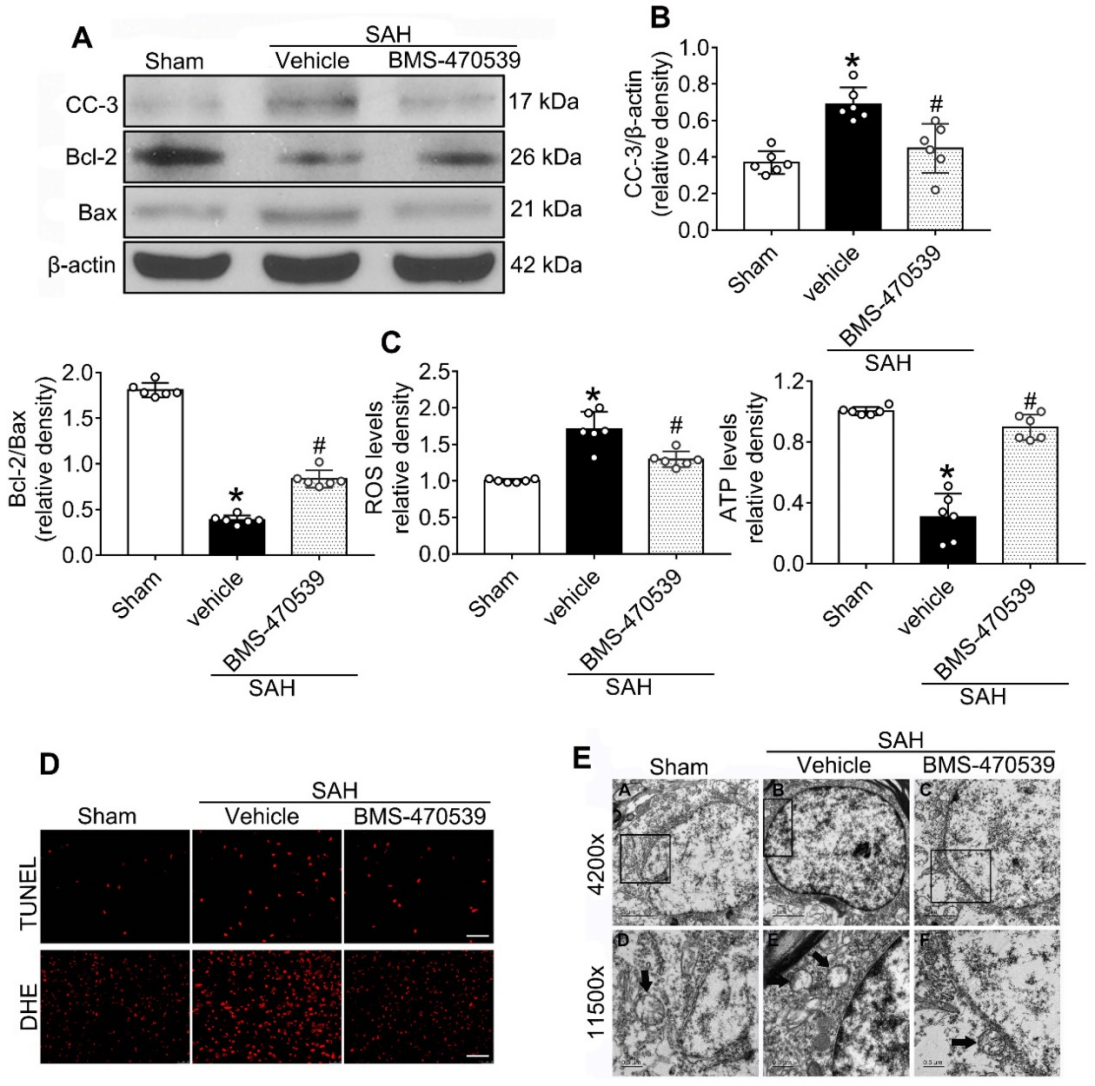
Neuroprotective effects of BMS-470539 on rats subjected to SAH. (A-B) Representative western blot images and quantitative analyses of cleaved caspase-3 (cc-3), Bcl-2 and Bax, n = 6 per group; (C) The levels of ROS and ATP, n = 6 per group; (D) Representative images of immunofluorescence staining of TUNEL and DHE, n = 4 per group, Scale bar = 50 μm; (E) Transmission electron microscopy images of the morphometric changes of the brain tissues in sham, SAH + vehicle, and SAH + BMS-470539 groups (scale bar = 2 μm for 4200 x; scale bar = 0.5 μm for 11500 x), n = 3 per group. The data were shown as means ± SD, and were compared using 1-way ANOVA followed by the Tukey post-hoc test. *p < 0.05 *vs.* sham, &*p <* 0.05 *vs.* SAH + vehicle.

**Figure 4:**
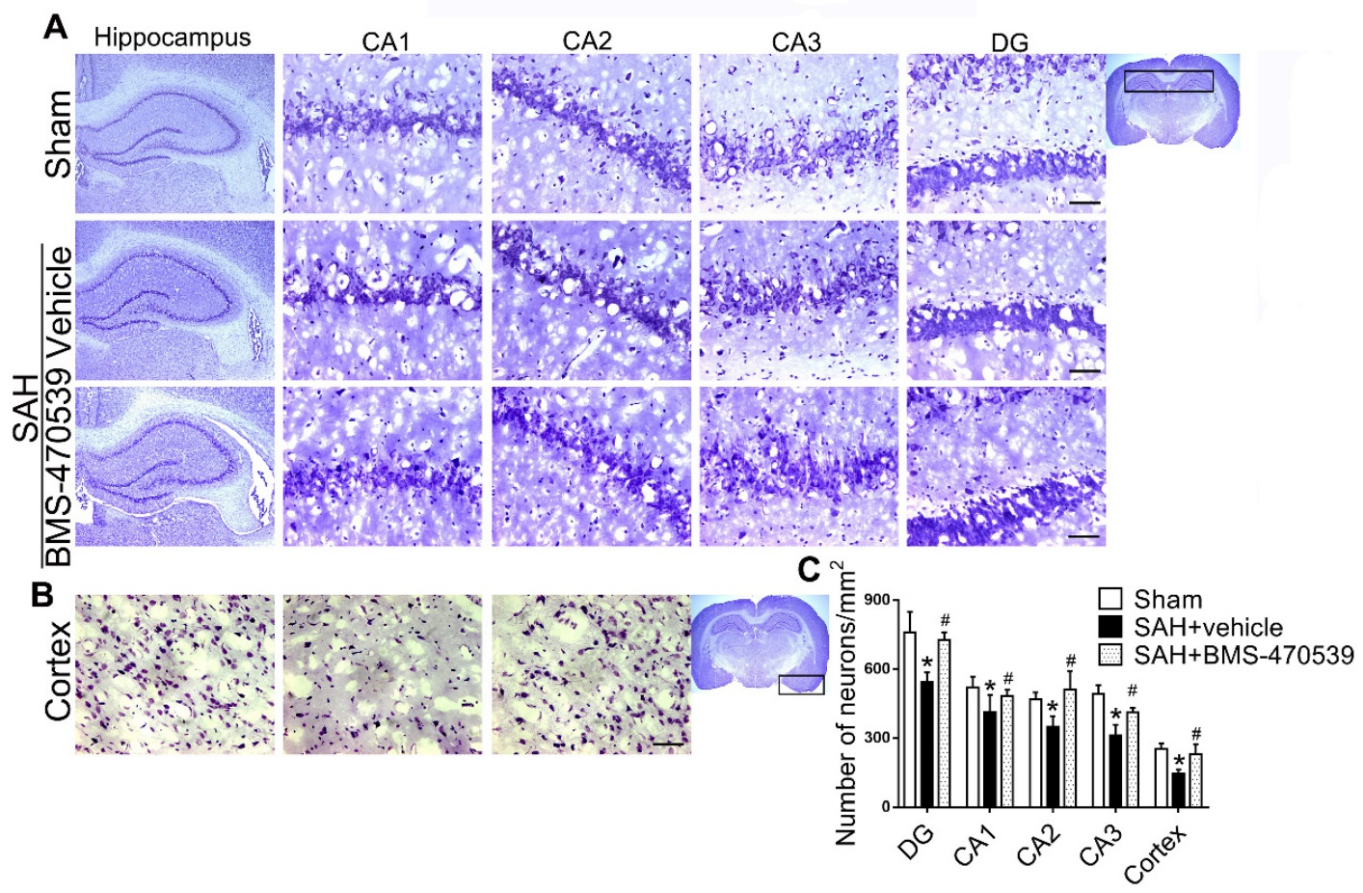
Representative images and quantitative analysis of Nissl staining of hippocampus (DG, CA 1, CA 2, and CA 3 areas) and cerebral cortex demonstrating reduced injury in BMS-470539 treated rats. Scale bar = 200 μm (general) and 50 μm (regions), n = 4 per group; The data were shown as means ± SD and compared by 2-way ANOVA test. *p < 0.05 *vs.* sham, &*p <* 0.05 *vs.* SAH + vehicle.

The results of western blot analysis showed that the administration of BMS-470539 significantly decreased the protein expression of cleaved caspase-3, but increased the ratio of Bcl-2/Bax when compared with the SAH group (P < 0.05; Figure 3A-B). Our results also indicated that BMS-470539 can decrease the levels of ROS-, TUNEL-, and DHE-positive cells, while increasing the ATP level (P < 0.05; Figure 3C-D). The Nissl staining that was performed to evaluate morphological changes of neurons in both the hippocampus and cerebral cortex in the long-term experiment. The results showed that neurons had sharp demarcation and rich cytoplasm in the sham group, whereas they had shrunken cell bodies and condensed nuclei in the SAH + vehicle group (Figure 4A-C). However, treatment with BMS-470539 markedly improved the morphological status when compared with the SAH + vehicle group (P < 0.05; Figure 4A-C). All results presented above demonstrated that BMS-470539 displays neuroprotective effects *via* the suppression of neuronal apoptosis and oxidative stress after SAH.

### Sub-organelle changes of neurons at 24 h after SAH

TEM was used to observe the sub-organelle changes of neurons, including the mitochondria and the nucleus, after the induction of SAH. In the sham group, intact mitochondria with prominent cristae were observed (Figure 3E). The nucleus was large and oval, with a clear nuclear membrane and homogenous distribution of chromatin. Conversely, in the SAH group, the heterogeneities of mitochondria and chromatin were obvious. The neurons had an irregular nuclear membrane, chromatin condensation, many vacuoles, and swollen mitochondria (Figure 3E). Treatment with BMS-470539 dramatically increased mitochondrial tubular networks and integrity. Chromatin condensation was reduced as well (Figure 3E).

### Inhibition of MC1R with MSG-606 abolished the neuroprotective effects of BMS-470539

**Figure 5:**
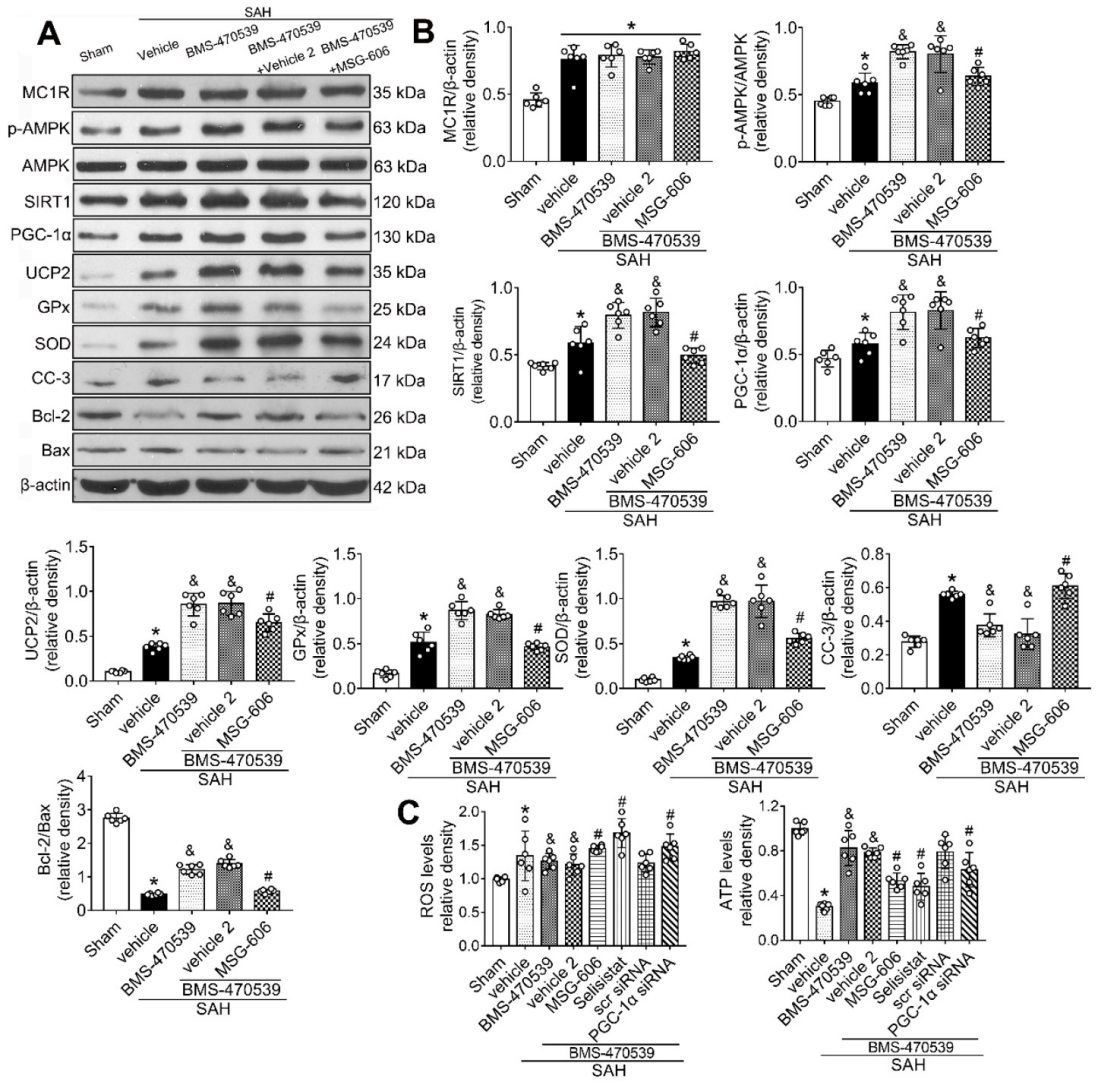
Inhibition of MC1R with MSG-606 abolished the neuroprotective effects of BMS-470539 at 24 h after SAH. (A) Representative western blot images; (B) Quantitative analyses of MC1R, p-AMPK, SIRT1, PGC-1α, UCP2, GPx, SOD, CC-3, Bcl-2, Bax; (C) ROS and ATP levels at 24 h after SAH. n = 6 for each group. Data were shown as means ± SD and compared by 1-way ANOVA followed by the Tukey post-hoc test. *p < 0.05 *vs.* sham, &*p <* 0.05 *vs.* SAH + vehicle, #*p <* 0.05 *vs.* SAH + BMS-470539.

To further explore the neuroprotective role of MC1R after SAH, we inhibited its functions. To achieve this, we used MSG-606, a specific inhibitor of MC1R. First, we assessed the neurological functions of rats in different groups. The rats that received BMS-470539 treatment, which showed significant improvement in neurobehavioral tests when compared with the vehicle treated group. However, this effect was abolished with the administration of MSG-606 (P < 0.05; Figure S3). Moreover, the results of western blotting indicated that the protein expression of p-AMPK, SIRT1, PGC-1α, UCP2, SOD, GPx, and Bcl-2 were significantly increased, whereas the levels of cleaved caspase-3 and Bax were reduced in rats that received treatment of BMS-470539 (*vs* SAH + vehicle, P < 0.05). We also tested ROS and ATP levels. The results revealed that the ROS level was increased, whereas the ATP level was decreased after the administration of BMS-470539 in rats subjected to SAH. However, the use of MSG-606 notably reversed these effects. Moreover, the levels of p-AMPK, SIRT1, PGC-1α, UCP2, SOD, GPx, Bcl-2, and ATP were decreased, but the levels of cleaved caspase-3, Bax, and ROS were increased at 24 h after SAH (P < 0.05; Figure 5A-C).

### SIRT1 was involved in MC1R-mediated neuroprotection

**Figure 7:**
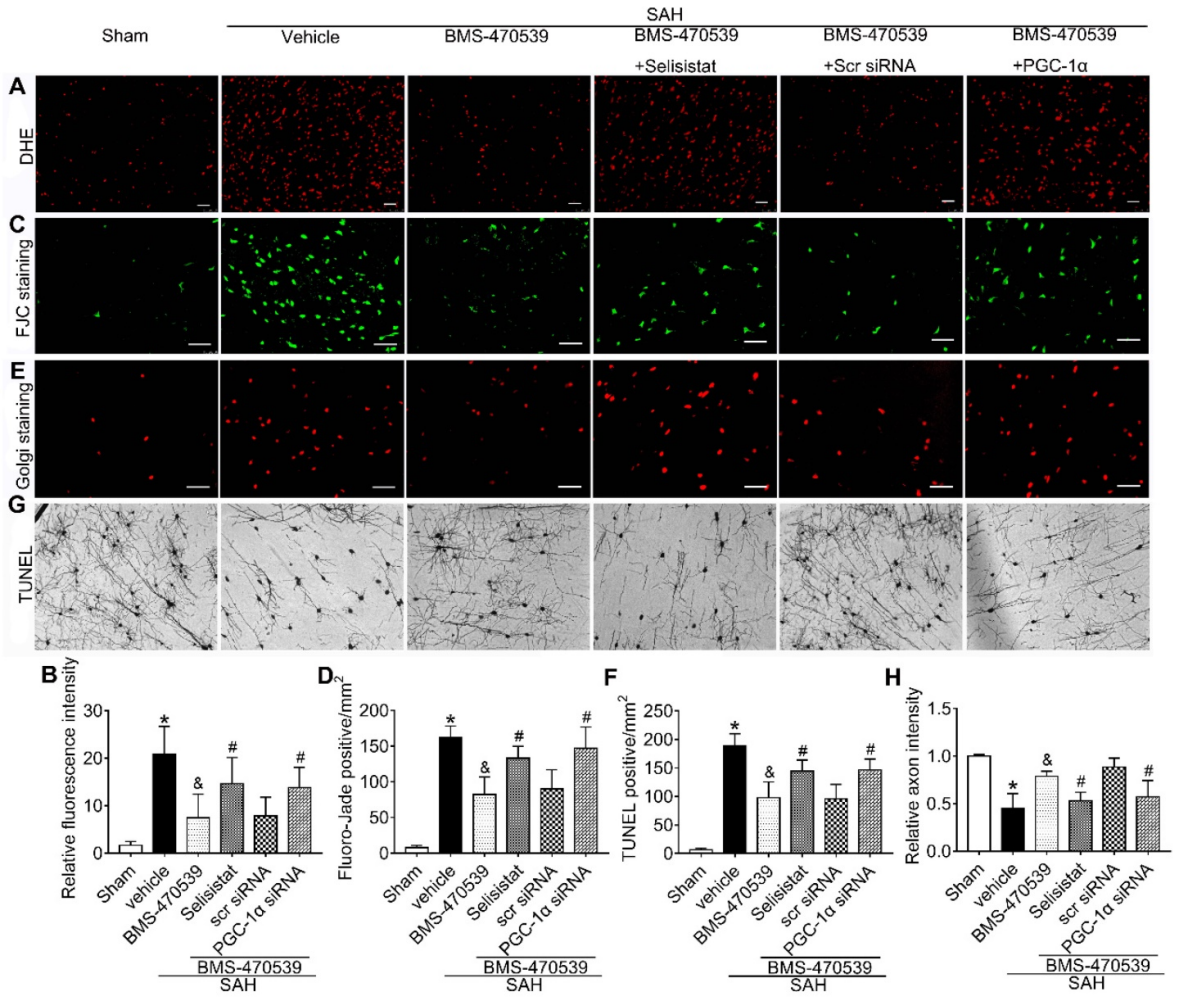
SIRT1 and PGC-1α were involved in MC1R-mediated neuroprotection at 24 h after SAH. Representative images and quantification of immunofluorescence and Golgi staining of (A, B) DHE; (C, D) FJC; (E, F) TUNEL; (G, H) Golgi. n = 4 for each group. Scale bar = 50 μm. Data were shown as means ± SD and compared by 1-way ANOVA, followed by the Tukey post-hoc test. *p < 0.05 *vs.* sham, &*p <* 0.05 *vs.* SAH + vehicle, #*p <* 0.05 *vs.* SAH + BMS-470539.

To verify the role of SIRT1 in MC1R-mediated neuroprotection, selisistat, a specific inhibitor of SIRT1, was administered. Initial neurobehavior assessment in different groups showed that selisistat significantly offset the neuroprotective effects of BMS-470539 after SAH (*vs* SAH + BMS-470539 + vehicle 2, P < 0.05; Figure S4). In addition, western blot analysis indicated that selisistat did not influence the protein expression levels of MC1R. However, the levels of p-AMPK, SIRT1, PGC-1α, UCP2, SOD, GPx, Bcl-2, and ATP were significantly decreased, while the levels of cleaved caspase-3, Bax and ROS were increased in the SAH + BMS-470539 + selisistat group compared to the SAH + BMS-470539 group (P < 0.05, Figure 5C, 6A-B). The results of immunofluorescence staining revealed that DHE- (Figure 7A-B), FJC- (Figure 7C-D), and TUNEL- (Figure 7E-F) positive cells were significantly increased after SAH, but this effect was abolished with the use of selisistat. Moreover, Golgi staining displayed notably increased integrity of axons after BMS-treatment; however, the number of broken axons was significantly increased by the use of selisistat (P < 0.05; Figure 7G-H). Consequently, all of the above results indicated that SIRT1 acted as a downstream factor of MC1R, and its inhibition abolished the anti-apoptotic and anti-oxidative stress effects of BMS-470539.

**Figure 8:**
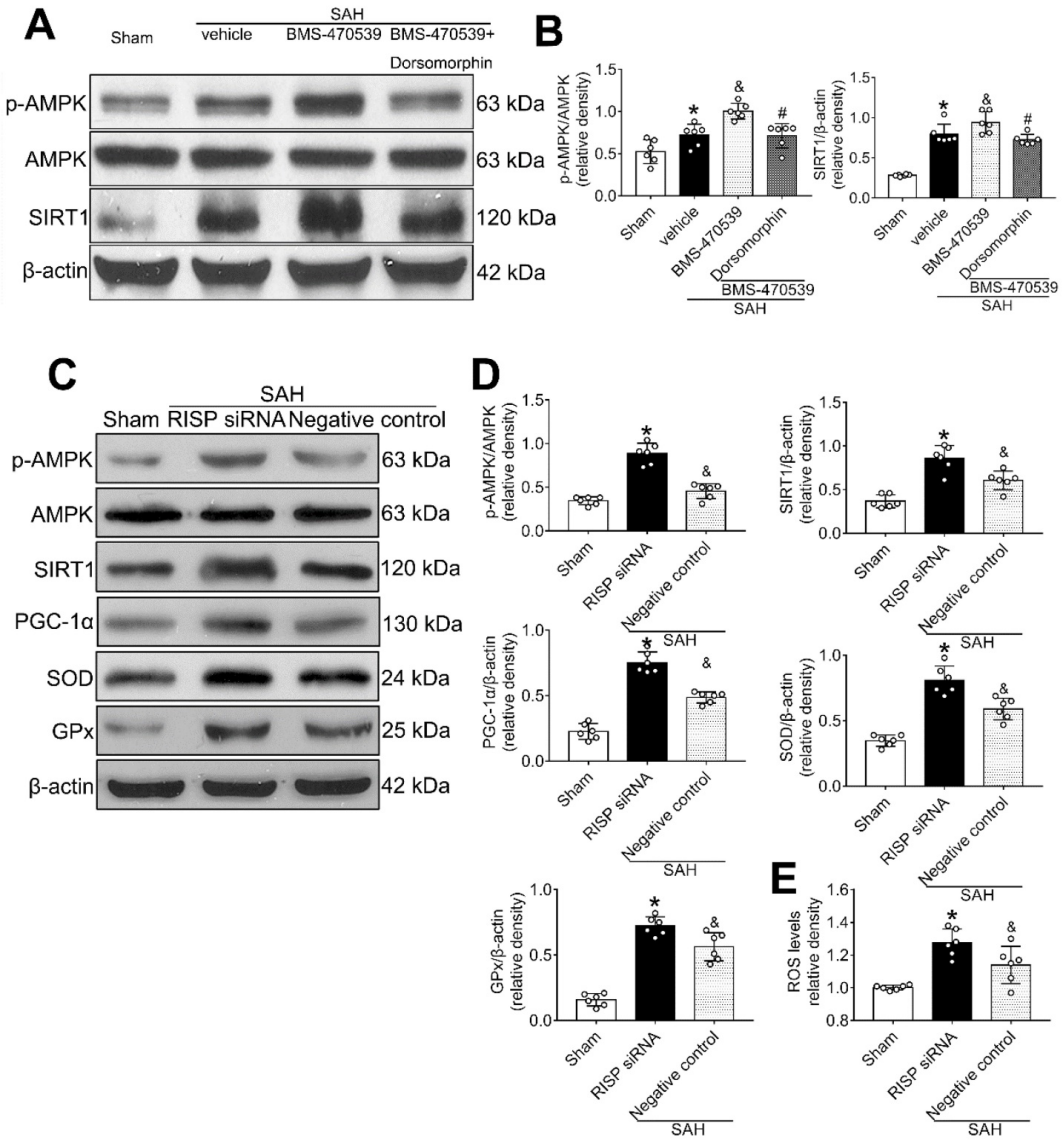
AMPK inhibitor decreased the levels of SIRT1, cellular apoptosis and oxidative stress at 24 h after SAH, which inversely stimulated the activation of AMPK. (A, B) Representative western blot images and quantitative analyses of p-AMPK and SIRT1; (C, D) Representative western blot images and quantitative analyses of p-AMPK, SIRT1, PGC-1α, GPx, SOD. n = 6 for each group; (E) Levels of ROS at 24 h after SAH. n = 6 for each group. Data were shown as means ± SD, and were compared using 1-way ANOVA followed by the Tukey post hoc test. *p < 0.05 *vs.* sham, &*p <* 0.05 *vs.* SAH + vehicle, #*p <* 0.05 *vs.* SAH + BMS-470539.

Then, we applied dorsomorphin, the AMPK inhibitor, to evaluate the relationship between AMPK and SIRT1. Western blot analysis showed that the levels of both p-AMPK and SIRT were decreased after the use of dorsomorphin, while the p-AMPK level was not influenced by selisistat, suggesting that SIRT1 expression was regulated by AMPK (P < 0.05, Figure 8A-B).

### Mitochondrial ROS reversely stimulated AMPK-dependent antioxidant responses

Rabinovitch *et al.* has reported that mitochondrial ROS is an intrinsic activator of AMPK, which can further stimulate the expression of antioxidant enzymes and reversely inhibit the production of mitochondrial ROS 40. To confirm this assertion in the setting of SAH, we used siRNA to silence the Rieske iron-sulfur protein (RISP), a component of complex III of the electron transport chain (ETC) that regulates ROS production from the mitochondria 41. The results showed that the level of ROS was significantly increased after the induction of SAH when compared with the sham group. Moreover, the levels of AMPK, SIRT1, PGC-1α, SOD, and GPx were all increased, suggesting the potential relationship between ROS and AMPK-dependent antioxidant responses. After knocking out RISP with siRNA, we found that the level of ROS was increased. Concurrently, the levels of AMPK, and its downstream proteins (SIRT1, PGC-1α, SOD and GPx), were further upregulated, confirming that the results of Rabinovitch are also applicable in the setting of SAH (P < 0.05, Figure 8C-E).

### Anti-oxidative stress and anti-apoptotic effects of BMS-470539 were partly abolished by PGC-1α siRNA at 24 h after SAH

The PGC-1α siRNA was applied to evaluate the role of PGC-1α in MC1R-mediated neuroprotective pathway after SAH. Treatment with PGC-1α siRNA markedly decreased the neurobehavior test scores (P < 0.05; Figure S5) after SAH, and also reduced the levels of PGC-1α, UCP2, SOD, GPx, Bcl-2, and ATP, but increased the levels of cleaved caspase-3, Bax, and ROS (P < 0.05, Figure 5C, 9A-B). DHE-, FJC-, and TUNEL-positive cells were also significantly upregulated after the use of PGC-1α siRNA. Moreover, PGC-1α siRNA reversed the protective effects of BMS-470539 on axons, and increased the number of injured axons. We did an additional experiment to test the depletion effect of APJ siRNA in naive rats, which indicated that APJ siRNA reduced the level of APJ by 60.7% (Figure S6).

### PGC-1α participated in MC1R-mediated neuroprotection *via* the suppression of mitochondrial fission

**Figure 10:**
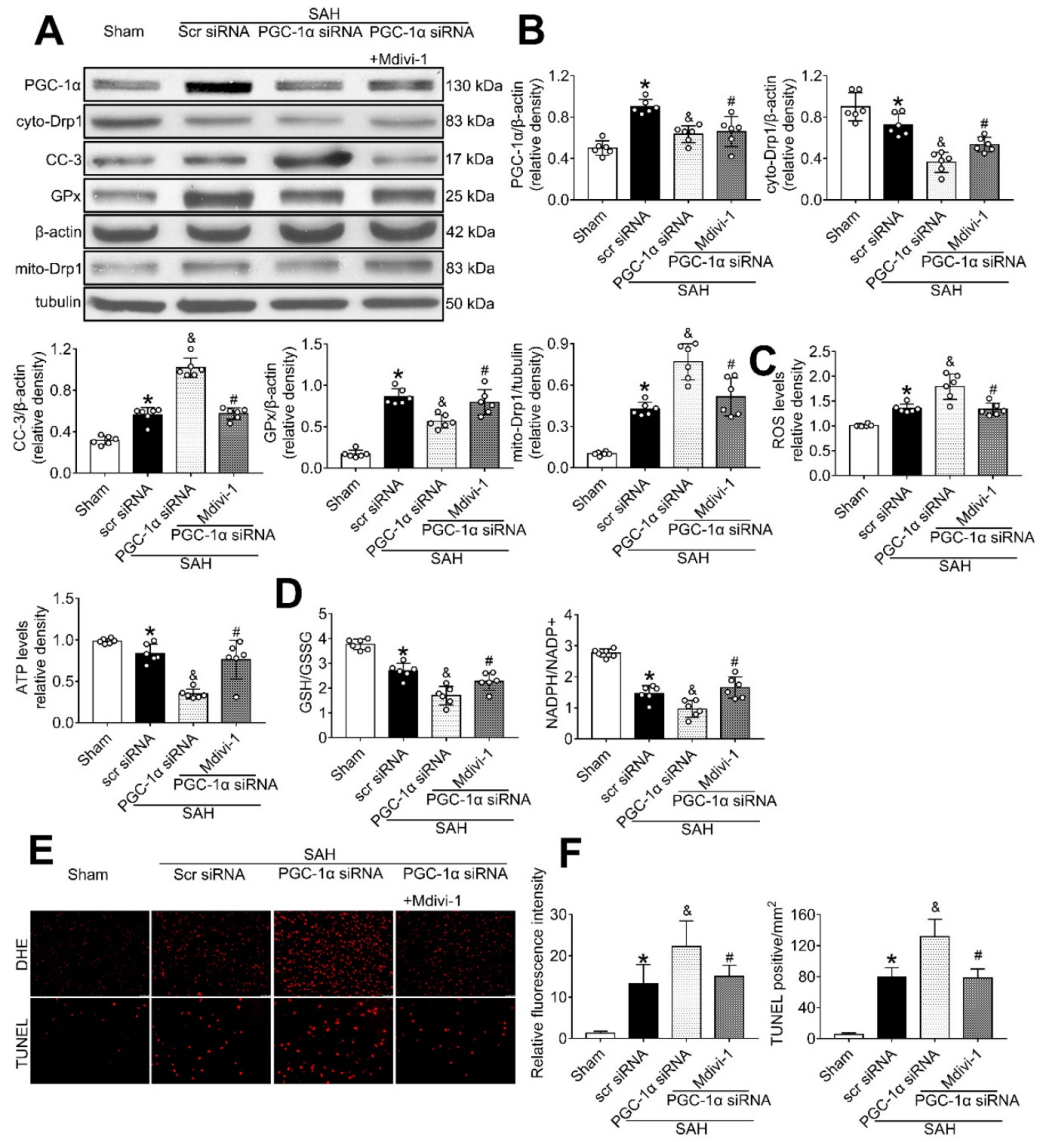
The effects of PGC-1α siRNA was partly mediated by the suppression of Drp1. (A) Representative western blot images; (B) Representative western blot images and quantitative analyses of PGC-1α, cyto-Drp1, CC-3, GPx, and mito-Drp1. n = 6 for each group; (C-D) Levels of ROS, ATP, GSH/GSSG ratio and NADPH/NADP^+^ ratio at 24 h after SAH. n = 6 for each group; (E-F) Representative images and quantitative analysis of immunofluorescence staining of TUNEL and DHE, n = 4 per group. Scale bar = 50 μm; Data were shown as means ± SD, and were compared using 1-way ANOVA followed by the Tukey post hoc test. *p < 0.05 *vs.* sham, &*p <* 0.05 *vs.* SAH + vehicle, #*p <* 0.05 *vs.* SAH + BMS-470539.

To determine whether PGC-1α can suppress mitochondrial fission, we tested the level of Drp1, a commonly used marker for mitochondrial fission. The results showed that PGC-1α siRNA alone decreased the levels of cyto-Drp1, GPx, and ATP, but increased the levels of cleaved caspase-3, mito-Drp1, and ROS (P < 0.05, Figure 10A-C). However, these effects were reversed by the use of Mdivi-1, the specific inhibitor of Drp1. Moreover, immunofluorescence staining results revealed that Mdivi-1 reduced DHE- and TUNEL-positive cells in the SAH+PGC-1α siRNA+Mdivi-1 group when compared with the SAH + PGC-1α group (P < 0.05; Figure 10E-F). We also assessed the GSH/GSSG and NADPH/NADP^+^ ratios to further evaluate the roles of PGC-1α and Drp1 in controlling ROS. The results showed that the administration of Mdivi-1 significantly reduced the GSH/GSSG and NADPH/NADP^+^ ratios when compared with the SAH + PGC-1α group (P < 0.05; Figure 10D). Consequently, the above results suggested that PGC-1α reduces ROS and apoptosis after SAH by preventing the translocation of Drp1 from the cytoplasm to the mitochondrial membrane.

## Discussion

The present study indicates that there is a novel mechanism of MC1R-mediated neuroprotection in a rat model of SAH, and functions by decreasing the oxidative stress and apoptosis through the AMPK/SIRT1/PGC-1α signaling pathway. The novel findings of this study were: (1) the protein expression of SIRT1 and PGC-1α were notably upregulated, and peaked at 24 h after SAH; (2) the administration of BMS-470539 significantly improved the short- and long-term neurobehavior test scores, including learning and memory abilities after SAH; (3) the administration of BMS-470539 significantly reduced the levels of ROS and apoptotic factors, but increased the levels of SIRT1, PGC-1α, and antioxidant enzymes; (4) the inhibition of MC1R with MSG-606 significantly abolished the anti-oxidative stress and anti-apoptotic effects of BMS-470539, and worsened the neurological deficits; (5) the selective SIRT1 inhibitor, selisistat, offset the positive effects of the BMS-470539/MC1R system by increasing the levels of ROS and pro-apoptotic factors at 24 h following SAH. (6) PGC-1α exerted neuroprotective effects by reducing mitochondrial fission induced by Drp1.

First, we determined the expression pattern of SIRT1 and PGC-1α at different time points. The results showed that the levels of these two proteins were significantly increased and peaked at 24 h after SAH. Next, we conducted double immunofluorescence staining to show the cellular localization of MC1R, which suggested that MC1R was mainly colocalized with neurons and microglia. The evidence above implied that there is a potential involvement of MC1R in the regulation of neuronal apoptosis.

MC1R, a polypeptide with seven α-helical transmembrane domains, has recently gained great attention due to its role in numerous important physiological processes, such as neuroprotection 42, 43. Schaible *et al.* has demonstrated that the activation of MC1R can significantly reduce brain injuries by reducing neuonal apoptosis in a mouse model of traumatic brain injury 44. In our earlier study, we reported that the activation of MC1R can consistently reduce brain injury by suppressing neuroinflammation following SAH. However, MC1R has a far greater role in the CNS than just the suppression of neuroinflammation. Instead, it has substantial effects on decreasing ROS and apoptosis, protecting DNA, etc. 45, 46. Therefore, more studies are necessary to comprehensively explore the effects of MC1R in the setting of SAH.

The spread of blood into the subarachnoid space after the induction of SAH is associated with severe brain edema and neurological deficits 47. The breakdown of blood cells results in the accumulation of toxic agents, and causes further oxidative stress and neuronal death, leading to long-term neurological deficits 48. Our study demonstrated that the activation of MC1R with its agonist, BMS-470539, notably alleviated neurological deficits after SAH. Moreover, the results of long-term neurobehavior assessment with rotarod and Morris water maze tests showed that the administration of BMS-470539 shortly after the onset of SAH demonstrated beneficial effects on long-term movement, coordination, memory, and spatial learning abilities. At the molecular level, we found that treatment with BMS-470539 provided neuroprotection by reducing oxidative stress and apoptosis, and by preserving mitochondrial integrity.

Then, we studied the underlying mechanisms behind MC1R-mediated anti-oxidative stress and anti-apoptotic effects. PGC-1α, as a transcriptional regulator protein, plays an important role in many metabolic activities. For example, PGC-1α can regulate the gluconeogenesis in liver cells, and it can also regulate thermogenesis of brown fat 49. In addition, PGC-1α has been found to have a crucial role in regulating mitochondrial function and oxidative stress in recent years. Chuang *et al.* has suggested that PGC-1α can increase the mitochondrial matrix and promote the formation of mitochondria 50. As a transcriptional regulator, PGC-1α can reduce ROS levels by promoting the production of antioxidant stress kinases, such as SOD, catalase, and GPx, but also by increasing the levels of mitochondrial membrane proteins, such as UCP2. In addition, PGC-1α may reduce ROS levels by regulating oxidative phosphorylation, and by fundamentally reducing the damage of ROS to cells 51, 52. PGC-1α plays a quite important role in the liver and heart tissues, as well as the CNS. For example, Ruan *et al.*^53^ has indicated that PGC-1α can improve the integrity of the blood-brain barrier, and reduce the damage induced by stroke. Research conducted by Robinson and Rasouri has shown that increased PGC-1α levels can maintain normal mitochondrial function, and improve neurological functions of patients with Alzheimer's and Parkinson's diseases 54, 55. In the current study, we have shown that the level of PGC-1α protein began to increase at 3 h after SAH, and reached the highest point at 24 h. This finding was similar to the changes in protein expression levels of MC1R and AMPK in previous reports, indicating that the change in PGC-1α level may be related to MC1R and AMPK.

Multiple studies have consistently reported that PGC-1α requires both AMPK phosphorylation and SIRT1 deacetylation to be fully activated 56. AMPK is a heterotrimeric protein that plays an important role in the regulation of cell energy metabolism. In our previous study, we confirmed that AMPK can manage neuroinflammation by regulating the TBK1/NF-κB signaling pathway. Many other studies have found that AMPK suppresses inflammation and plays an important role in cellular oxidative stress and apoptosis. For example, Park *et al.* has suggested that *Sipjeondaebo-tang* can reduce oxidative stress by activating the CaMKK2/AMPK signaling pathway, thereby reducing liver damage 57; In addition, Gui *et al.* has reported that Salidroside can inhibit smooth muscle by regulating the AMPK/mTOR/ULK1 signaling pathway. It should be noted that ROS increases the expression of AMPK as well 58. SIRT1 is a member of the Sirtuin family, and is widely expressed in various tissues, such as in the liver, muscle, adipose, brain, etc. 59, 60. Similar to AMPK, the level of SIRT1 increases or decreases based on the alterations in the level of nutrient metabolism of the body, and the changes in its protein level mainly depend on the changes in the level of NAD + and NAD + / NADH ratio. Several studies have reported that AMPK can indirectly increase the activity of SIRT1 by regulating the activity of NAD +, which can further deacetylate and activate PGC-1α 61, 62. In line with this, we found that the levels of PGC-1α, and its downstream antioxidant kinases, were significantly reduced, while intracellular ROS and apoptosis were increased with the use of a SIRT1 inhibitor.

However, after knocking out PGC-1α using siRNA, the levels of AMPK and SIRT1 did not change notably; however, the downstream antioxidant stress kinase was decreased. Therefore, our results indicate that the anti-oxidative stress and anti-apoptotic effects of PGC-1α are regulated by its upstream AMPK/SIRT1 signaling pathway. In addition, we used siRNA to silence RISP to verify whether intrinsic ROS can retroactively activate AMPK in the setting of SAH. The results showed that upregulation of mitochondrial ROS significantly increased the level of AMPK-dependent antioxidant responses, further highlighting the important role of AMPK in maintaining cellular redox equilibrium.

In line with the above-mentioned evidence, Torrens *et al.* has reported that MC1R can exert its anti-oxidative stress and anti-apoptotic effects by regulating the level of PGC-1α 26. Besides, how MC1R regulates the level of PGC-1α remains unclarified. In our previous study, we found that MC1R can exert its downstream effects by regulating AMPK 25. Conversely, the anti-oxidative stress and anti-apoptotic effects of PGC-1α were regulated by its upstream AMPK/SIRT1 signaling pathway. Therefore, we believe that MC1R regulates oxidative stress and neuronal apoptosis by regulating the AMPK/SIRT1/PGC-1α signaling pathway.

This article mainly explores the specific mechanism of MC1R's anti-oxidation and anti-apoptosis, but there are several shortcomings: (1) we applied the pharmacological agonist or antagonist of targeted proteins in this study; however, we were unable to prove the effects of activation or inhibition in some cases, nor could we exclude off-target effects, which may decrease the reliability of the results; (2) the brain is a complex network consisting of various cell types. Although we focused on neurons in this study, other cell types, such as microglia or astrocytes, may affect our results; (3) anti-oxidative stress and anti-apoptotic effects of MC1R have multiple pathways; however, in this work we have evaluated only the AMPK / SIRT1 / PGC-1α signaling pathway; (4) although this article indicated that AMPK changes the activity of SIRT1 by regulating the level of NAD +, the exact underlying mechanism remains to be unveiled and needs further experiments to verify this pathway; (5) in addition, the underlying mechanisms of how PGC-1α inhibited the activity of Drp1 is still unclear. Therefore, the results in this study should be cautiously interpreted before further experimental methods are introduced. We would like to address these issues and conduct more in-depth exploration in our future studies.

## Conclusion

BMS-4705 39 reduces the level of oxidative stress in cells by inducing the activity of MC1R, and by regulating the AMPK/SIRT1/PGC-1α signaling pathway after SAH. Consequently, treatment with BMS-4705 39 diminishes mitochondrial fission to maintain normal mitochondrial function, thereby reducing oxidative stress and neuronal apoptosis, and improving neurological functions following SAH.

## Supplementary Material

Supplementary figures, tables and procedures.

## Figures and Tables

**Figure 6:**
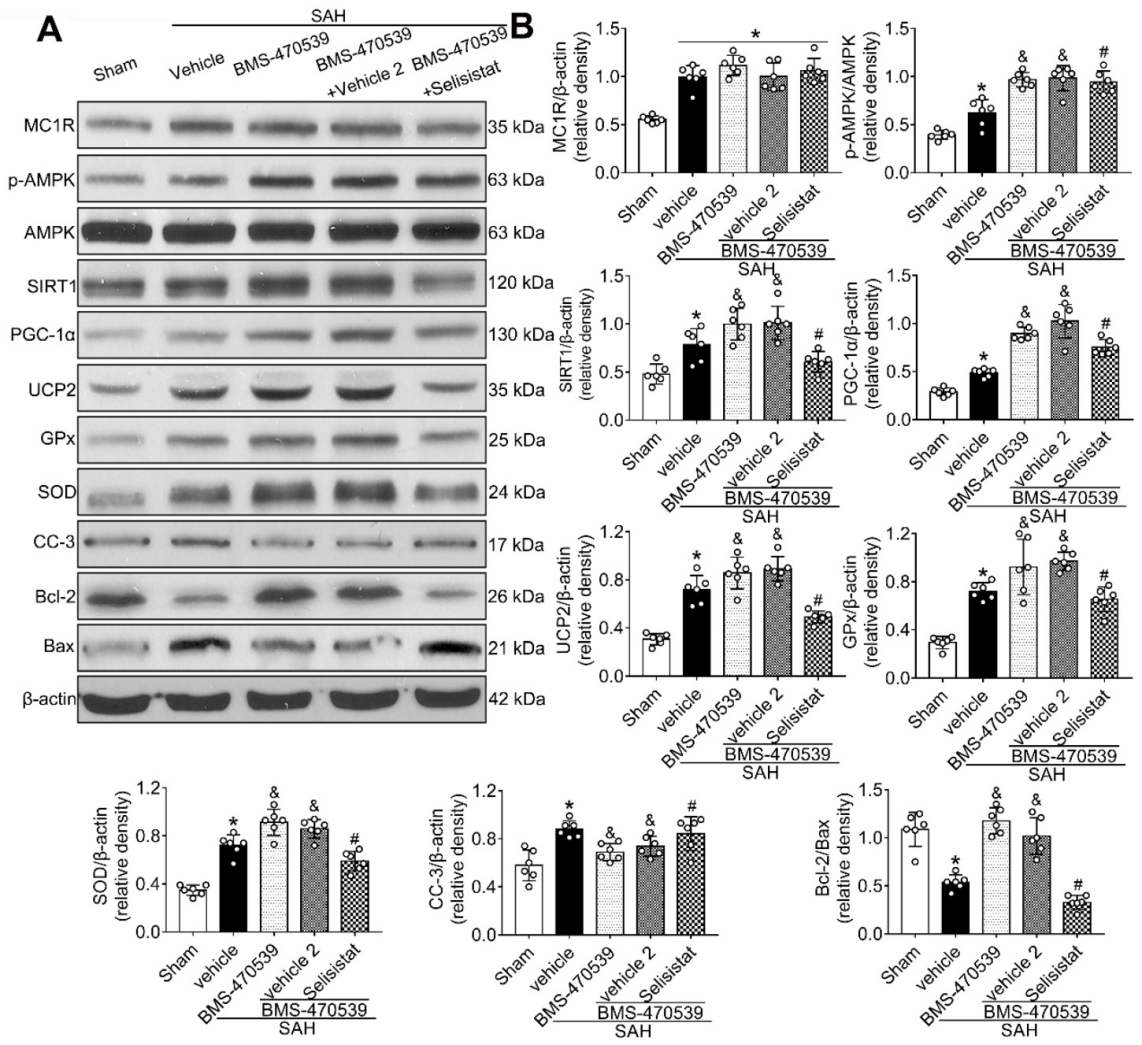
SIRT1 inhibitor, selisistat reversed the anti-oxidative stress and anti-apoptotic effects of MC1R at 24 h after SAH. (A) Representative western blot images; (B) Quantitative analyses of MC1R, p-AMPK, SIRT1, PGC-1α, UCP2, GPx, SOD, CC-3, Bcl-2, Bax. Data were shown as means ± SD and compared by 1-way ANOVA followed by the Tukey post-hoc test. *p < 0.05 vs. sham, &p < 0.05 vs. SAH + vehicle, #p < 0.05 vs. SAH + BMS-470539.

**Figure 9:**
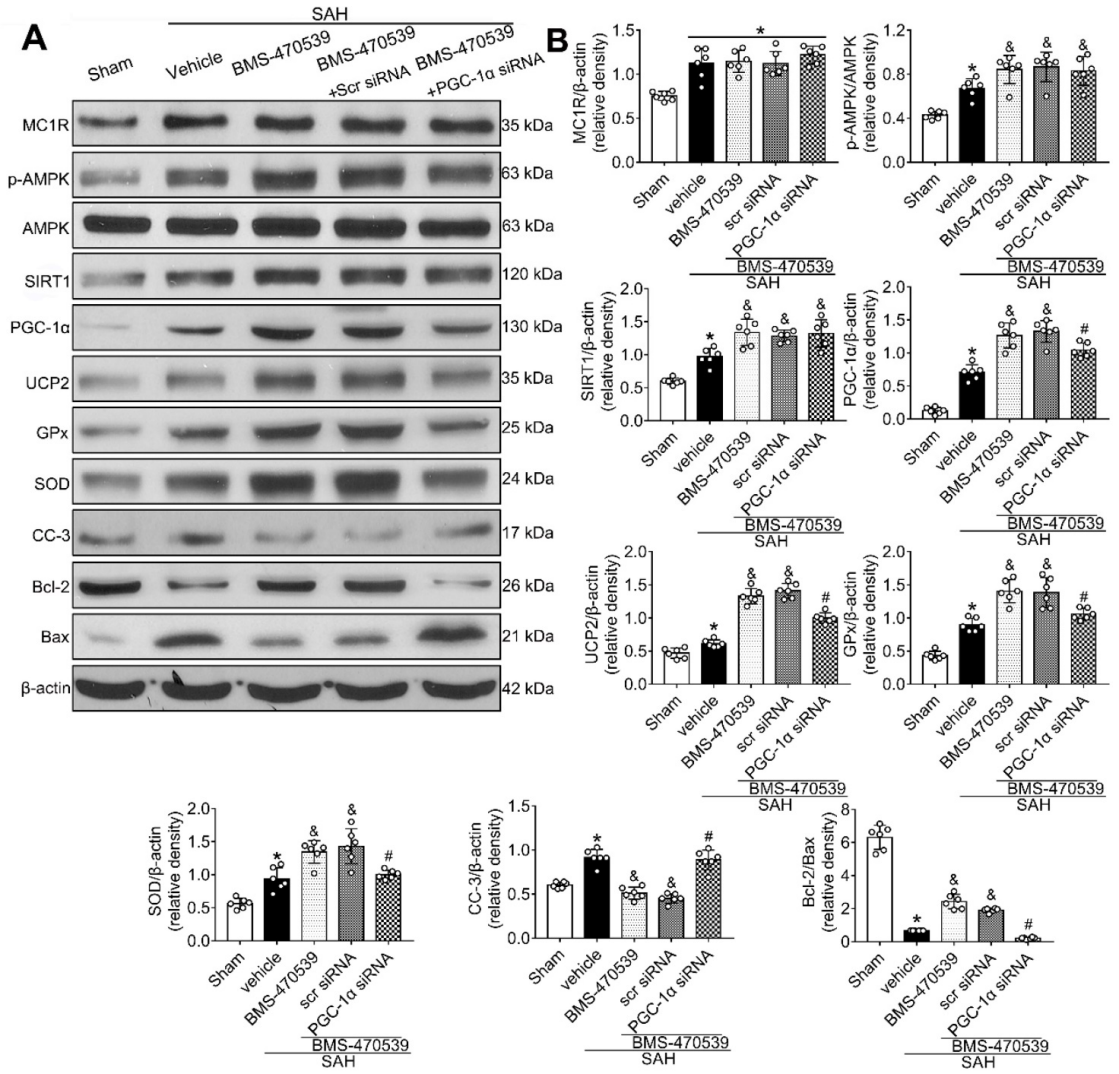
Knockout of PGC-1α with siRNA aggravates oxidative stress and apoptosis following SAH. (A) Representative western blot images; (B) Quantitative analyses of MC1R, p-AMPK, SIRT1, PGC-1α, UCP2, GPx, SOD, CC-3, Bcl-2, Bax. Data were shown as means ± SD, and were compared using 1-way ANOVA followed by the Tukey post hoc test. *p < 0.05 *vs.* sham, &*p <* 0.05 *vs.* SAH + vehicle, #*p <* 0.05 *vs.* SAH + BMS-470539.

## Abbreviations

EBI: early brain injury
SAH: subarachnoid hemorrhage
MC1R: melanocortin 1 receptor
CNS: central nervous system
ROS: reactive oxygen species
PGC-1α: peroxisome proliferator-activated receptor gamma coactivator 1-alpha
SOD: superoxide dismutase
GPx: glutathione peroxidase
AMPK: Adenosine 5'-monophosphate (AMP)-activated protein kinase
SIRT1: silent information regulator 1
TEM: transmission electron microscopy
GSH/GSSG: glutathione/oxidized glutathione
GFAP: glial fibrillary acidic protein
TUNEL: terminal deoxynucleotidyl transferase-dUTP nick end labeling
FJC: Fluoro-Jade C
DHE: Dihydroethidium
RISP: Rieske iron-sulfur protein
ETC: electron transport chain
