# Supplementary material for: Melanocortin 1 receptor attenuates early brain injury following subarachnoid hemorrhage by controlling mitochondrial metabolism via AMPK/SIRT1/PGC-1α pathway in rats
Source: Theranostics. 2021 Jan 1;11(2):522–39. doi: 10.7150/thno.49426 (PMC7738864; doi:10.7150/thno.49426)
Supplement: Supplementary file 1 — Supplementary figures, tables and procedures. [file thnov11p0522s1.pdf]

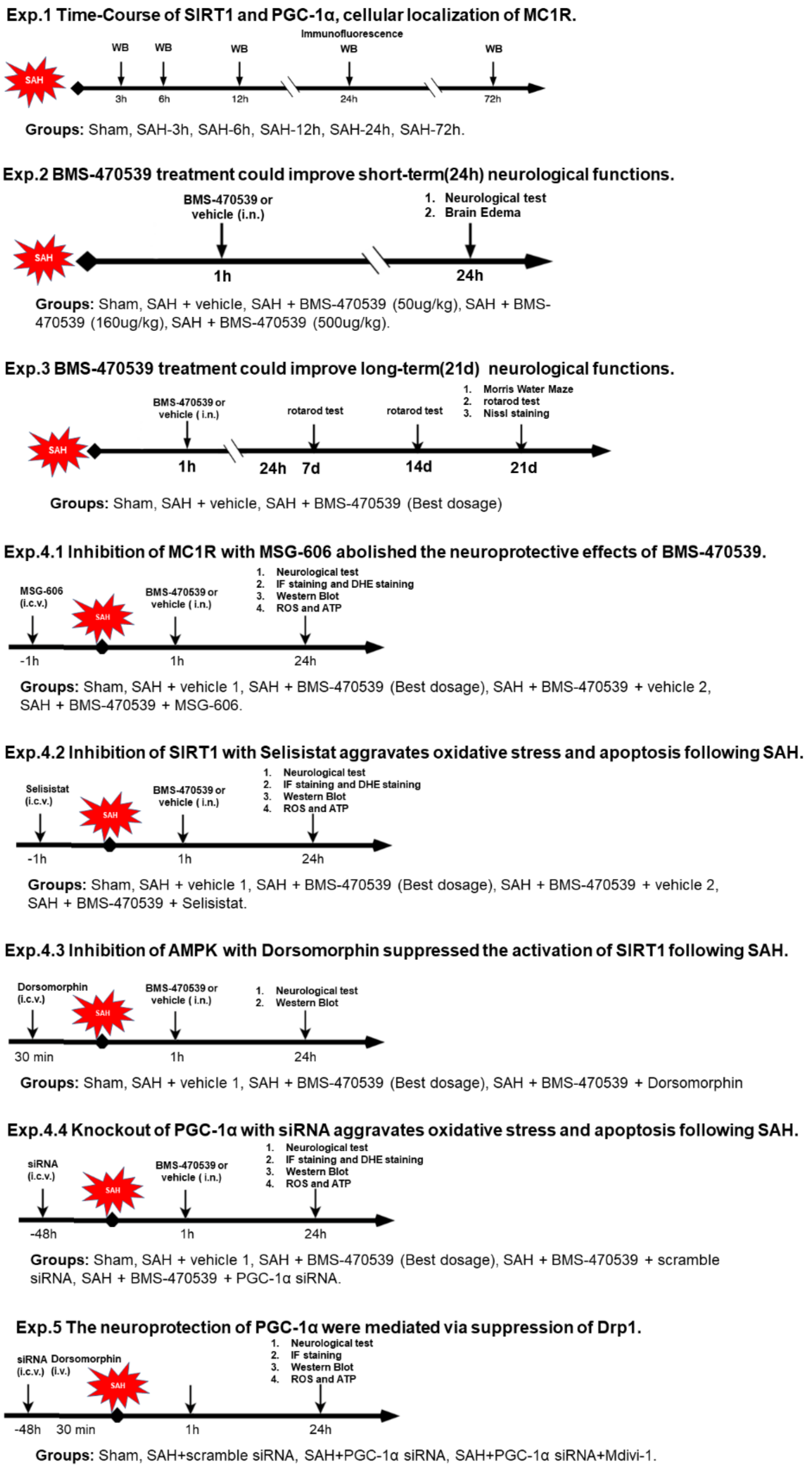

Figure S1. Experimental design of the study.

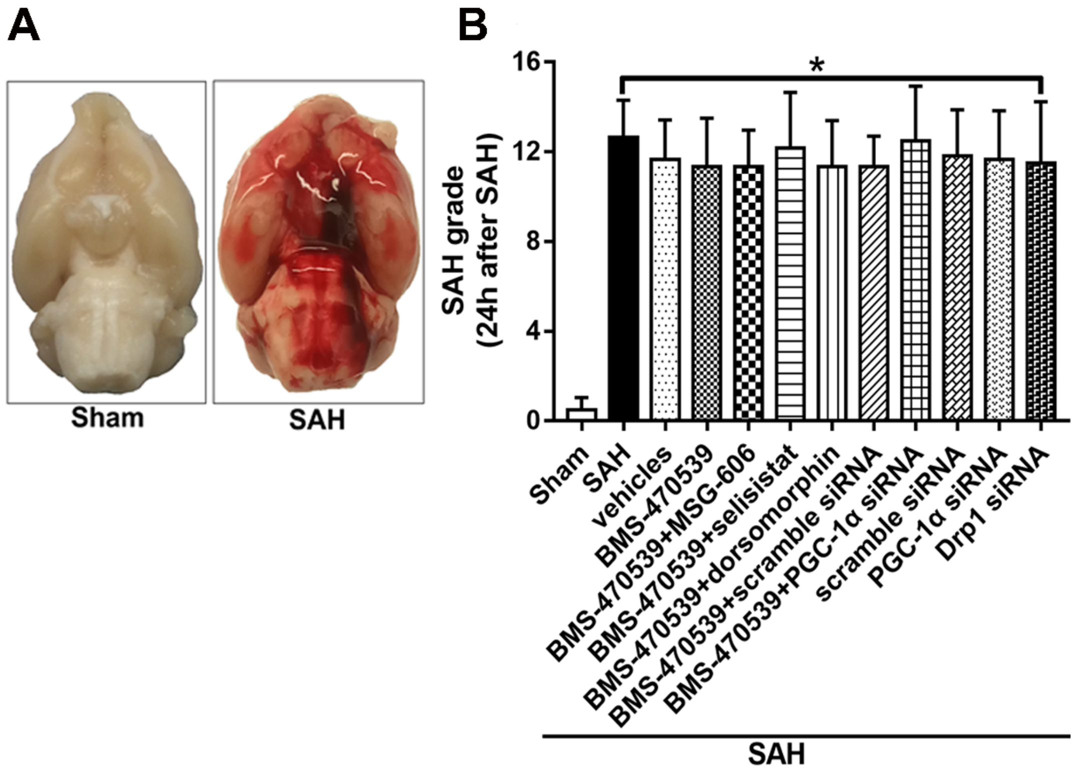

Figure S2. (A) Representative pictures of sham and SAH model; (B) SAH grade, Data were shown as means ± SD, and were compared by 1-way ANOVA followed by the Tukey post-hoc test. *p < 0.05 *vs.* sham.

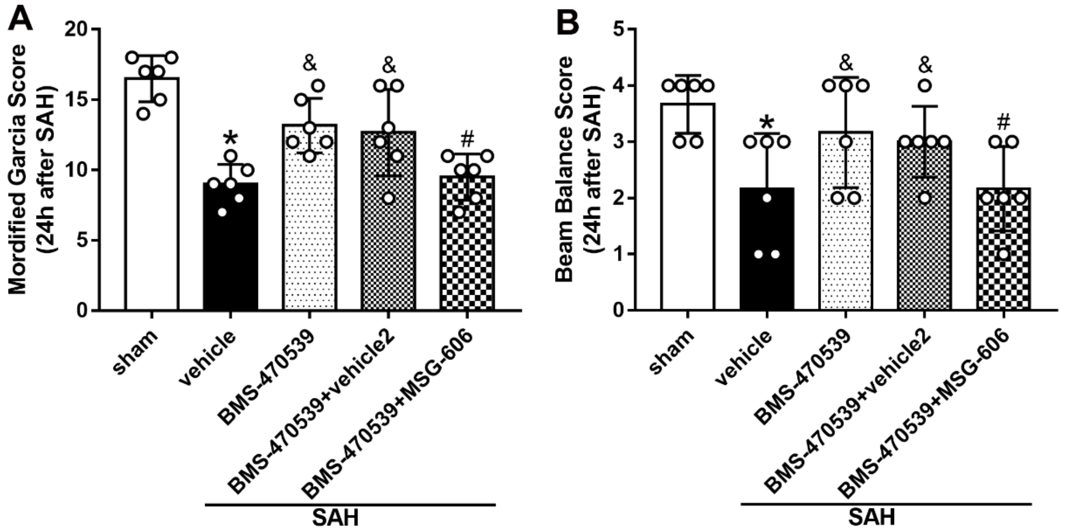

Figure S3. Inhibition of MC1R with MSG-606 abolished the neuroprotective effects of BMS-470539 at 24 h after SAH. Modified Garcia (A) and Beam balance scores (B). n = 6 for each group. Data were shown as means ± SD. *p<0.05 versus sham,

&p<0.05 versus SAH + vehicle, #p<0.05 versus SAH + BMS-470539.

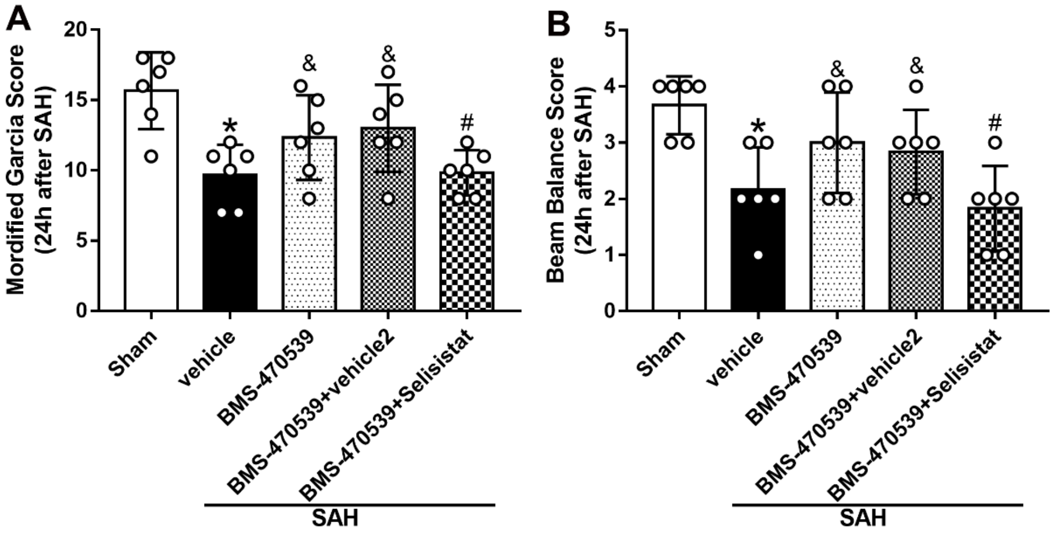

Figure S4. SIRT1 inhibitor, selisistat, reversed the anti-oxidative stress and anti-apoptotic effects of MC1R at 24 h after SAH. Modified Garcia (A) and Beam balance scores (B). n = 6 for each group. Data were shown as means ± SD. *p<0.05 versus sham, &p<0.05 versus SAH + vehicle, #p<0.05 versus SAH + BMS-470539.

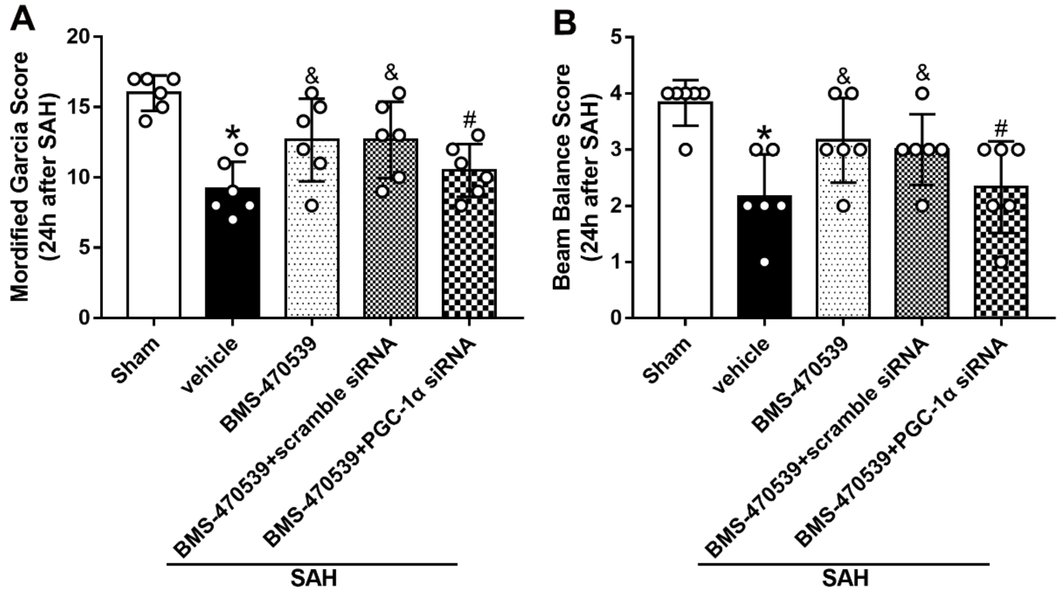

Figure S5. Knockout of PGC-1α with siRNA aggravated oxidative stress and apoptosis following SAH. Modified Garcia (A) and Beam balance scores (B). n = 6 for each group. Data were shown as means ± SD. *p<0.05 versus sham, &p<0.05

versus SAH + vehicle, #p<0.05 versus SAH + BMS-470539.

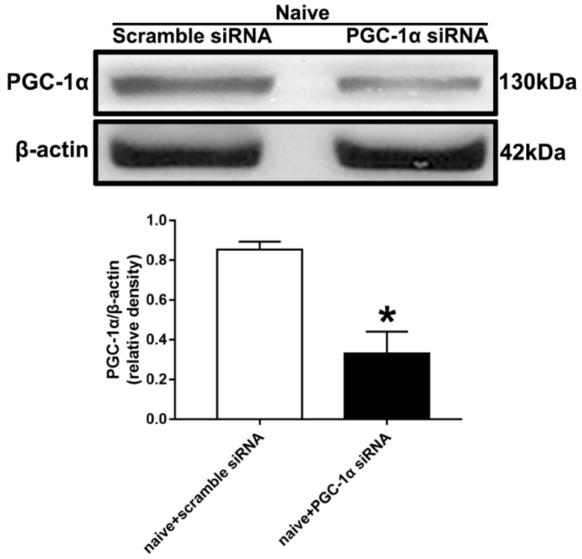

Figure S6. The use of PGC-1α siRNA significantly reduced the level of PGC-1α. (A) Representative Western blot images and quantitative analyses of PGC-1α. n = 3 for each group. The bars represent the mean ± SD. *P < 0.05 versus naive + scramble siRNA.

Table S1. Distribution of animals according to different groups.

Groups	Mortality	Excluded	Shared
**Exp. 1**			
Sham	0% (0/8)	0	0
SAH (3, 6, 12, 24, 72 h)	11.1% (4/36)	1	0
**Exp. 2**			
Sham	0% (0/6)	0	0
SAH+Vehicle	14.3% (1/7)	1	0
SAH+BMS-470539 (50ug/kg)	0% (0/6)	0	0
SAH+BMS-470539 (160ug/kg)	14.3% (1/7)	0	0
SAH+BMS-470539 (500ug/kg)	0% (0/6)	0	0
**Exp. 3**			
Sham	0% (0/29)	0	0
SAH+Vehicle	17.1% (6/35)	2	0
SAH+BMS-470539(Best dosage)	9.4% (3/32)	0	0
**Exp. 4.1**			
Sham	0% (shared)	0	16
SAH+Vehicle 1	0% (shared)	0	16
SAH+BMS-470539	0% (shared)	0	16
SAH+BMS-470539+vehicle 2	14.3% (2/14)	1	0
SAH+BMS-470539+MSG-606	7.7% (1/13)	0	0
**Exp. 4.2**			
Sham	0% (shared)	0	6
SAH+Vehicle 1	0% (shared)	0	6
SAH+BMS-470539	0% (shared)	0	6

SAH+BMS-470539+vehicle 2	0% (shared)	0	6
SAH+BMS-470539+selisistat	15.8% (3/19)	0	0
**Exp. 4.3**			
Sham	0% (shared)	0	6
SAH+Vehicle 1	0% (shared)	0	6
SAH+BMS-470539	0% (shared)	0	6
SAH+BMS-470539+Dorsomorphin	14.3% (1/7)	1	0
**Exp. 4.4**			
Sham	0% (shared)	0	6
SAH+Vehicle 1	0% (shared)	0	6
SAH+BMS-470539	0% (shared)	0	6
SAH+BMS-470539+scramble siRNA	11.1% (2/18)	1	0
SAH+BMS-470539+PGC-1α siRNA	15.8% (3/19)	0	0
**Exp. 5**			
Sham	0% (shared)	0	16
SAH+scramble siRNA	11.1% (2/18)	1	0
SAH+PGC-1α siRNA	15.8% (3/19)	1	0
SAH+PGC-1α siRNA+Drp1 inhibitor	5.9% (1/17)	0	0
**Exp. 6**			
naïve+scramble siRNA	0% (0/3)	0	0
naïve+PGC-1α siRNA	0% (0/3)	0	0
**Total**			
**naïve**	**0% (0/6)**		
**Sham**	**0% (0/43)**		
**SAH**	**11.83% (33/273)**	**9**	**124**

SAH, subarachnoid hemorrhage; PGC-1α, peroxisome proliferator-activated receptor gamma coactivator 1-alpha.

Table S2. Neurological Evaluation: Modified Garcia score.

Test	Score			
	**0**	**1**	**2**	**3**
Spontaneous Activity (in cage for 5 min)	No movement	Barely moves position	Moves but does not approach at least three sides of cage	Moves and approaches at least three sides of cage
Spontaneous movements of all limbs	No movement	Slight movement of limbs	Moves all limbs but slowly	Move all limbs same as pre-SAH
Movements of forelimbs (outstretching while held by tail)	No outreaching	Slight outreaching	Outreach is limited and less than pre-SAH	Outreach same as pre-SAH
Climbing wall of wire cage		Fails to climb	Climbs weakly	Normal climbing
Reaction to touch on both side of trunk		No response	Weak response	Normal response
Response to vibrissae touch		No response	Weak response	Normal response

SAH, subarachnoid hemorrhage;

Table S3. Neurological Evaluation: Beam balance

	0	1	2	3	4
Beam walking (60 sec)	No walking and falls off	No walking but remains on beam	Walking but falls off	Walking less than 20 cm	Walking beyond 20 cm

Table S4. Plans of Morris water maze test.

	**Day 1**		**Day 2**	**Day 3**	**Day 4**	**Day 5**	**Day 6**
	**Platform location**	**Starting direction**	**Platform Location: SW Starting Location as follows**				**No platform**
**Trial 1**	SW	S	W	N	N	E	N
**Trial 2**	NW	N	S	W	E	S	
**Trial 3**	NE	E	N	E	W	W	
**Trial 4**	SE	W	E	S	S	N	

S, south; N, north; E, east; W, west.

Table S5. Physiological data of the rats after surgeries (dead and excluded animals are not included).

Group	Rats used	T (°C)	HR(/min)	BP (mmHg)	BG (mmol/L)	PO2(mmHg)	PCO2 (mmHg)
Sham	43	37.5±0.18	366±4.3	132±0.21	6.4±0.29	76±2.8	38±2.1
SAH	32	37.6±0.21	359±8.8	131±0.89	6.6±0.49	73±2.5	39±1.6
SAH+vehicles	35	37.8±0.16	353±7.0	129±0.98	6.5±0.37	74±3.4	40±2.5
SAH+BMS-470539 (50ug/kg)	6	38.0±0.22	365±6.4	132±1.45	6.8±0.56	73±2.9	39±2.7
SAH+BMS-470539 (160ug/kg)	35	37.5±0.15	362±8.0	135±1.37	6.5±0.47	75±3.1	38±4.3
SAH+BMS-470539 (500ug/kg)	6	37.8±0.18	361±5.6	130±0.99	6.7±0.52	73±3.5	40±2.6
SAH+BMS-470539+vehicles	12	37.7±0.15	360±7.3	131±0.88	6.5±0.34	75±4.2	39±1.7
SAH + BMS-470539 + MSG-606	12	37.4±0.16	359±6.4	130±1.15	6.6±0.56	74±1.9	41±2.9

SAH + BMS-470539 + selisistat	16	37.6±0.16	360±8.3	133±0.89	6.7±0.36	76±4.3	41±1.8
SAH + BMS-470539 + dorsomorphin	6	37.6±0.21	365±6.6	135±0.87	6.5±0.37	74±5.1	39±2.5
SAH + BMS-470539 + scramble siRNA	16	37.1±0.25	357±7.2	127±0.72	6.4±0.49	75±3.6	42±2.8
SAH + BMS-470539 + PGC-1α siRNA	16	37.8±0.15	354±6.3	130±0.90	6.6±0.51	72±5.1	40±2.5
SAH + scramble siRNA	16	37.5±0.20	355±8.1	133±0.96	6.5±0.39	77±5.1	41±2.7
SAH + PGC-1α siRNA	16	37.6±0.19	364±7.9	133±1.25	6.7±0.48	74±4.3	40±2.6
SAH + PGC-1α siRNA + Mdivi-1	16	37.5±0.17	358±6.1	129±0.82	6.5±0.34	75±3.9	39±2.5

SAH, subarachnoid hemorrhage; PGC-1α, peroxisome proliferator-activated receptor gamma coactivator 1-alpha.

Supplemental Detailed procedures

SAH animal model

The rats that received anesthesia with 5% isoflurane were intubated and then positioned supine on a ventilator. The rats were kept under maintenance anesthesia with 3% isoflurane together with 65/35% medical air/oxygen during the surgery. Rats were closely monitored for heart and respiration rates throughout the procedure. We exposed the carotid artery and its bifurcation. Afterwards, a 4-0 sharpened nylon suture was inserted from the external carotid artery. The suture then went along the internal carotid artery and finally reached the bifurcation of the anterior and middle cerebral arteries, where a perforation was performed.

Intracerebroventricular injection

The rats were anesthetized with 5% isoflurane, and remained under anesthesia as described above. Next, we used a drill to make a burr hole 1 mm posterior to the bregma and 1.5 mm right lateral to the midline, at which point the drug was slowly administered (3.5 mm in depth, 0.5 μl/min). The needle was kept in place for 5 minutes, and then was slowly withdrawn over a period of 5 minutes. Finally, the burr hole and

incision were closed with bone wax and sutures, respectively. All surgical procedures were conducted with sterile techniques.

Morris water maze

Rats were required to find the submerged platform and the procedure was recorded by an overhead camera linked to a computer tracking system. We recorded their swim path, escape latency, and swim distance individually. The probe trial was performed on the last day of the test. During this test the platform was removed, and the duration of time spent in probe quadrant was recorded.

Sample collection

For the short-term outcomes study (24h after SAH), we chose the cortical region (x: 1 mm to 5 mm from midline; y: from bregma 5.16 mm to bregma -3.12 mm) according to "The Rat Brain In Stereotaxic Coordinates" (Fifth edition. Paxinos and Watson, 2005). The reasons are: 1) mordified Garcia and beam balance tests evaluate both motor and sensory functions of rats. The areas we chose for this study includes the primary and secondary motor and sensory cortex; 2) after the induction of SAH, the blood spreads mostly around the cortex. Therefore, we chose five different slices in this area for immonufluorescence staining, which can represent the brain injuries of motor and sensory cortex; 3) the expression level of MC1R is homogeneous in the selected area. Thus, the area we chose for this study is reliable and acceptable for studying the relationship between the neurological functions and molecular pathophysiological changes.

For long-term spatial learning and memory study, we dissected hippocampus to further study according to a previous report by Hagihara et al. (Hagihara et al. 2009).

Detailed steps:

a. In a deeply anesthetized rat, the brain was carefully dissected out from the skull and placed into ice-cold phosphate-buffered saline (PBS).

b. In a Petri dish containing ice-cold PBS, the brain was cut along the longitudinal fissure of the cerebrum using a surgical knife, and the regions posterior to lambda (midbrain, hindbrain, and cerebellum) were cut off.

c. The cerebral hemisphere was placed medial side up and using forceps, the diencephalon (thalamus and hypothalamus) was carefully removed under a dissection microscope. This exposed the medial side of the hippocampus, allowing for visualization of the dentate gyrus which is distinguishable from Ammon's horn by the gaps between them, avoiding injury to the hippocampus or surrounding area that would make it more difficult to isolate the dentate gyrus.

d. A sharp needle-tip (e.g., 27-gauge needle) was inserted into each side of the dentate gyrus (boundaries of the dentate gyrus and Ammon's horn), and the needles were slided superficially along the septo-temporal axis of the hippocampus to isolate the dentate gyrus.

e. The isolated dentate gyrus was picked up using needle or forceps and placed in a sample tube. The thus-obtained dentate gyrus tissue sample was used immediately or stored in a deep-freezer for later use.

f. The dentate gyrus was isolated from the other cerebral hemisphere using the same method.

Western blot analysis.

After being anesthetized, the rats received trans-cardiac perfusion (0.1 M PBS). The ipsilateral/left cerebral cortex was then sampled for western blotting. In brief, 40 μg of protein from each sample underwent electrophoresis, and then the protein was transferred onto nitrocellulose membranes (100 V, 80 minutes). Afterwards, the membranes were incubated with the following primary antibodies overnight at 4°C: MC1R (1:500, ThermoFisher, PA5-75342), p-AMPK (1:1000, Cell Signaling, #2535), AMPK (1:2000, Cell Signaling, #5832), SIRT1 (1:5000, Abcam ab109272), PGC-1α (1:1000, Abcam ab54481), UCP2 (1:500, Abcam ab67241), GPx (1:500, Abcam ab59524), SOD (1:500, Abcam ab9722),

cleaved caspase-3 (1:500, Abcam ab49822), Bcl-2 (1:500, Abcam ab59348), Bax (1:3000, Abcam ab32503), Drp1 (1:1000, Abcam ab184247), and β-actin (1: 3000, Santa Cruz sc-47778). Secondary antibodies were then applied at room temperature for 1 h. Finally, the bands were probed with ECL Plus chemiluminescence reagent Kit (Amersham Biosciences, Arlington Heights, PA), and visualized using an imaging system (Bio-Rad, Versa Doc, model 4000). Next, Image J software (NIH) was used to measure intensity. The results were displayed as relative density (grayscale value of the target proteins /β-actin or total proteins). During western blot quantification, when proteins were detected, beta-actin of the same sample derived from the same membrane, corresponding with the proteins were detected simultaneously as well. Therefore, all proteins were normalized with their own beta-actin from the same sample.

Immunofluorescence staining

After being anesthetized with 5% isoflurane, the rats received trans-cardiac perfusion with 0.1M PBS, followed by 10% formalin. Afterwards, we quickly collected the brains and placed them in 10% formalin (4°C, 24 h). The formalin was replaced with 30% sucrose solution for 3 days. The brains were cut into 10 μm sections. The slices were then fixed on slides and used for immunofluorescence staining. The brain slices were incubated at 4°C overnight with the following primary antibodies: MC1R (1:50, ThermoFisher, PA5-75342), Iba-1 (1:100, Abcam ab48004), glial fibrillary acidic protein (GFAP) (1:100, Abcam ab53554), and NeuN (1:100, Abcam ab104224). Secondary antibodies were then applied at room temperature (21 °C) for 2 h and the samples were assessed with a fluorescence microscope (Leica Microsystems, Germany).

Nissl staining

The rats were treated as described above for immunofluorescence staining. The collected brains were then cut into 16 μm slices. The slices were immersed with 0.5 % cresyl violet (Sigma-Aldrich, St. Louis, MO, USA) solution and dehydrated with 100% alcohol. Next, the sections were

washed with xylene and sealed with a coverslip. Finally, the sections were observed under a light microscope by a blinded investigator.

Golgi staining

Briefly, the rat brain tissues were removed quickly under deep anesthesia on day 28 after SAH. The freshly dissected brains were immersed in solution A and B for 2 weeks at room temperature and transferred into solution C for 72 h at 4 °C. The brains were sliced using a cryostat system (CM3050S; Leica Microsystems, Bannockburn, III, Germany) at a thickness of 100 μm. The following staining steps were completed according to the FD Rapid GolgiStain™ Kit manufacturer's protocol (Columbia, USA). Brightfield images for brain samples were obtained using an Olympus BX51 microscope (Olympus, Waltham, USA). Microphotographs were analyzed using Image Pro Plus 6.0 software (Media-Cybernetics, Bethesda, MD, USA).

Transmission electron microscopy

The rats received trans-cardiac perfusion with 0.1 M PBS and 4 % paraformaldehyde (pH = 7.4) after anesthetization. Then the perihematomal tissues were collected and grained into 1 mm^3 slices. Next, the slices were immersed into glutaraldehyde (2.5%) at 4°C overnight. Then, we put the samples in 1% osmium tetroxide for 1 h and dehydrated the samples with a series of graded ethanol. The tissues were immersed into a mixture of propylene oxide and resin (1:1). Four hours later, the samples were imbedded in resin. After that, we cut the samples in to 100nm sections and stained them with 4% uranyl acetate (20 min) and 0.5% lead citrate (5 min). Finally, the transmission electron microscopy (Philiphs Tecnai 10) was used to observe the ultrastructure of brain tissues.
